# Single-cell RNA sequencing reveals the intercellular crosstalk and the regulatory landscape of stromal cells during the whole life of the mouse ovary

**DOI:** 10.1093/lifemedi/lnae041

**Published:** 2024-12-28

**Authors:** Wan Jiang, Wenya Sun, Yue Peng, Hao Xu, Haonan Fan, Xin Jin, Yue Xiao, Yuxiang Wang, Pin Yang, Wenjie Shu, Jing Li

**Affiliations:** Bioinformatics Center of AMMS, Beijing 100850, China; State Key Laboratory of Reproductive Medicine and Offspring Health, Nanjing Medical University, Nanjing 210029, China; State Key Laboratory of Reproductive Medicine and Offspring Health, Nanjing Medical University, Nanjing 210029, China; Bioinformatics Center of AMMS, Beijing 100850, China; State Key Laboratory of Reproductive Medicine and Offspring Health, Nanjing Medical University, Nanjing 210029, China; Wuxi Maternity and Child Health Care Hospital, Affiliated Women’s Hospital of Jiangnan University, Wuxi 214000, China; The First Affiliated Hospital of Zhejiang University School of Medicine, Center of Reproductive Medicine, Hangzhou 310009, China; Bioinformatics Center of AMMS, Beijing 100850, China; School of Basic Medical Sciences, Anhui Medical University, Hefei 230022, China; Bioinformatics Center of AMMS, Beijing 100850, China; State Key Laboratory of Reproductive Medicine and Offspring Health, Nanjing Medical University, Nanjing 210029, China

**Keywords:** single-cell RNA sequencing, intercellular crosstalk, ovarian aging, stromal cell, mouse ovary

## Abstract

The heterogeneity of ovarian mesenchymal/stromal cells has just been revealed in both mice and humans. However, it remains unclear about the cellular development trace and the intercellular communication network in the whole life of the ovary. In the study, we integrated ours and published single-cell RNA sequencing data from E11.5 (embryonic day 11.5) until M12 (12-month-old) ovaries to show the dynamics of somatic cells along the developmental timeline. The intercellular crosstalk among somatic cell types was depicted with collagen signaling pathway as the most outgoing signals from stromal cells. We identified mesenchymal progenitor cells (CD24^+^) as the origin of stromal cells. Although their numbers decreased significantly in adults, the cells served as the major signal sender until ovarian senescence. Moreover, the ovarian injury could activate these stem cells and induce stroma remodeling in the aged ovary. Thus, mesenchymal progenitor cells may represent a new strategy to delay ovarian aging in the future.

## Introduction

In mammals, the reproductive capability is determined by the ovarian development, which provides a limited oocyte pool established during fetal life [1]. The follicle, composed of an inner oocyte with surrounding somatic cells and formed before or just after birth in humans or mice, is the basic unit of the ovary. In mouse embryos, ovarian development is initiated with a “bipotential” genital ridge, whereas oocytes are originated as primordial germ cells (PGCs) from extraembryonic mesoderm that proliferate and migrate into the genital ridge at E10.5 (embryonic day 10.5). The bipotential genital ridge then undergoes sex determination to be ovary at E11.5, where the localized female germ cells proliferate and associate with each other as syncytia or germ cell cysts between E10.5–13.5. Within the cyst, germ cells enter the prophase of meiosis in an asynchronous manner around E13.5–14.5 and at this stage, the cyst together with somatic pre-granulosa cells are called “ovigerous cords.” Starting from E16.5, with the meiosis arrest at the diplotene stage of meiosis I, germ cell cysts begin to breakdown, and pre-granulosa cells move in surrounding the oocyte to form primordial follicles [2–4]. As the ovarian reserve, finite primordial follicles should be maintained dormancy until being activated by surrounding stimuli [1, 4]. Then, the follicles develop through primary, secondary, and antral stages until preovulatory follicles. At this stage, response to preovulatory gonadotropin surges, ovulation occurs, and the remaining theca and granulosa cells transform to form the corpus luteum. Through the initial recruitment of primordial follicles and cyclic recruitment of antral follicles, follicles develop to either ovulate or undergo degeneration (atresia) until the depletion of the primordial pool which leads to the reproductive senescence [5].

During past decades, the vast majority of studies on ovarian biology have focused on folliculogenesis. From germline development, ovarian formation to oogenesis, folliculogenesis, and ovarian development, each of these stages is governed by a unique set of molecules and regulators. Moreover, it also requires coordinated interactions and communications between different cell types in the ovary. According to the ovarian structure, ovarian follicles comprise the ovarian parenchyma, whereas the components of the ovary that are not ovarian follicles are referred to as mesenchyme/stroma [6]. In addition to general cell types, such as immune cells, blood vessels, and nerve or lymphatic vessels, the ovarian stroma also contains ovarian epithelial cells or incompletely characterized stromal cells which include fibroblast-like, spindle-shaped, or interstitial cells. These cells participate in scaffolding the ovary structure and supporting follicular development by secreting extracellular matrix (ECM) proteins, such as collagen, laminin, or fibronectin. Recently, with the development of single-cell sequencing technologies, somatic cell components in the human ovarian inner cortex have been identified with different types of granulosa cells, thecal and stromal cells. According to the cell origins from stroma or follicles at different developmental stages, they divided five clusters of thecal and stromal cells with differential gene expressions [7]. However, in another single-cell analysis of the human ovarian cortex, the authors classified most ovarian somatic cells (83%) as stromal cells and when the data are integrated with the previous single-cell profiling of the human ovary, they found a close relationship between theca cells and the general stromal cells [8]. One single-cell transcriptomic study of ovarian aging in nonhuman primate ovaries identified stromal cells with specifically expressed TCF21 and COL1A2, while the authors found the oxidative damage to early-stage oocytes and granulosa cells is the crucial factor in ovarian functional decline with age [8]. As we know, the distribution and subtypes of stromal cells will likely differ according to their locations in the ovary. They will also be affected by follicle growth, ovulation, corpus luteum formation, or even ovarian aging process. One study generated a single-cell atlas during mouse ovarian development by collecting single cells from E10.5–P5 (postnatal day 5). Although they found the subtypes of mesenchymal populations, the authors focus on the two distinct pathways of pregranulosa cell differentiation and two waves of follicle development [9]. Another single-cell sequencing of the cycling murine ovary revealed the dynamics of cell states and cell types during the estrous cycle. In the study, the mesenchymal cell subtypes have been divided in detail as early theca, steroidogenic theca, smooth muscle cells, pericytes, and two interstitial stromal cell clusters: steroidogenic cells and fibroblast-like cells [10]. However, they did not mention how these cells coordinate with other cell types in the profound tissue remodeling during the process. It seems that with the development of the single-cell sequencing technique, although the ovarian cell types and transcriptome dynamics have been well described, the stromal cells and their regulatory role in the ovary are often neglected. Moreover, most studies focused on the ovary at different developmental stages or just revealed a static framework in the ovary. It lacks a global view of the dynamics of somatic cell types and cell states during the whole life of the ovary.

In this study, we sequenced 54,099 single cells (mainly ovarian somatic cells) from P14 (postnatal day 14), W8 (8-week-old), M8 (8-month-old), and M12 (12-month-old) mice by 10x Genomics technology and tried to establish a single-cell atlas of the ovary lifecycle by integrating public data generated by the same technique from E11.5 (embryonic day 5) to P5 (postnatal day 5) ovaries. We studied the landscape of cell–cell interactions and explored the cell types, signaling pathways, and regulatory mechanisms in mouse ovaries across the development. Our work reconstructed the cellular diagram from gonadal differentiation to primordial follicle formation until ovarian aging and delineated the regulatory roles of ovarian somatic cells, especially mesenchyme stromal cells during the whole life of the ovary.

## Results

### Single-cell RNA-seq of mouse ovary

**Figure 1.**
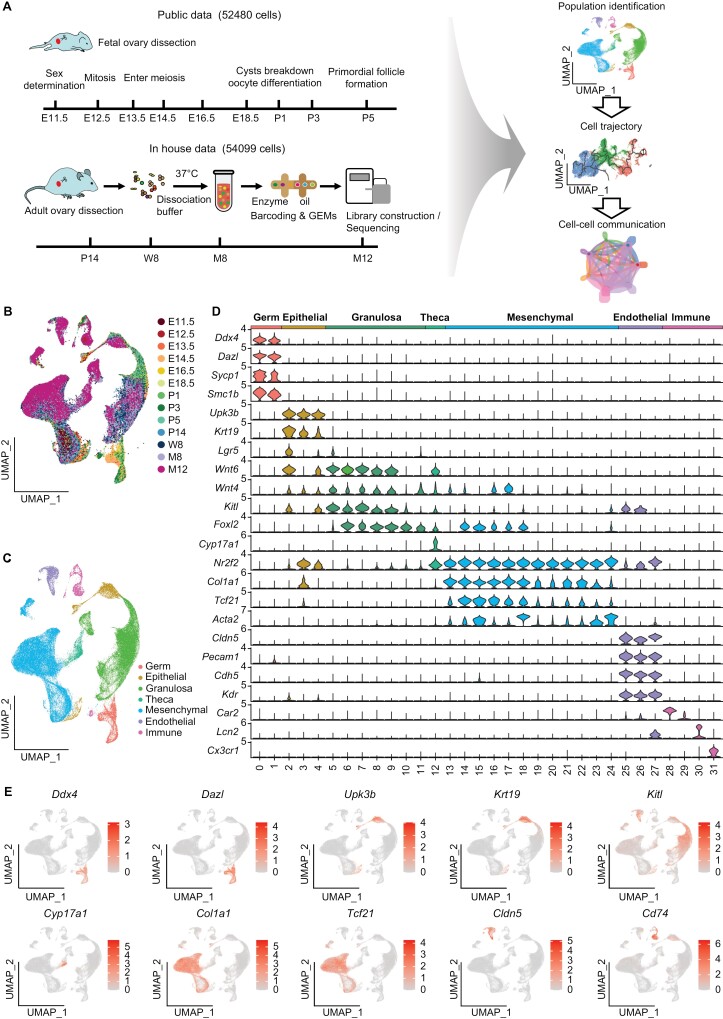
**Single-cell RNA sequencing reveals cell types during the whole life of mouse ovary.** (A) The work flow of data collection, processing, and analysis. Mouse ovaries were collected at P14, W8, 8M, and 12M for scRNA-seq by 10x Genomics Chromium system. The scRNA-seq data from E11.5–P5 ovaries were obtained publicly and integrated together for the following analysis including cell clustering and identification, pseudo-time analysis, and cell–cell communication analysis. The figure was created using materials from the SciDraw website. (B) UMAP plot of the single cell distribution in the mouse ovary, colored by developmental stage from E11.5 to M12. (C) UMAP plot of the single cell distribution in the mouse ovary, colored by the identified 7 cell types. (D) Violin plot of marker gene expression in each cell population, colored by the identified 7 cell populations. *X* axis: cluster number. *Y* axis: normalized gene expression. (E) UMAP visualizations of marker gene expression of each cell population. Germ cells: *Ddx4*, *Dazl*; epithelial cells: *Upk3b*, *Krt19*; granulosa genes: *Kitl*; Theca: *Cyp17a1*; mesenchymal cells: *Col1a1*, *Tcf21*; endothelial cells: *Cldn5*; immune cells: *Cd74*.

To decipher the landscape of cell–cell interactions in mouse ovary across development, we collected ovaries at P14 (prepuberty stage), W8 (young), M8 (fertility decreased stage), and M12 (aged stage) for 10× Genomics scRNA-seq and integrated the data with previous scRNA-seq data from fetal gonads (E11.5–E18.5) and newborn ovaries (P1–P5) for a global analysis (see Methods) [9, 11, 12]. Using a unified single-cell analysis pipeline (Fig. 1A and Methods), we obtained in total 106,579 cells from the ovaries of all samples, including 52,480 cells from public data and 54,099 cells from in-house data. The median number of cells per sample was 8198 cells, with a median of 9105 unique molecular identifiers corresponding to 2939 genes per cell (Table S1). We integrated these high-quality cells into a unified dataset after batch correction (Fig. S1A and S1B). We clustered all cells using uniform manifold approximation and projection (UMAP) [13] (Figs. 1B, 1C and S1C) and obtained 7 major cell types with 31 cell clusters based on the expression of canonical marker genes (Figs. 1D, 1E, S1E and S1F; Table S2). These cells include germ cells (cluster 0–2) that highly expressed *Ddx4* and *Dazl* [14, 15], epithelial cells (cluster 2–4) with *Upk3* [16] and *Krt19* [17] expression, granulosa cells (cluster 5–11) that expressed *Wnt6*, *Wnt4*, *Kitl*, and *Foxl2* [18–20], theca cells (cluster 12) with *Cyp17a1* [21] expression, mesenchymal cells (cluster 13–24) that highly expressed *Nr2f2*, *Col1a1*, *Tcf21*, and *Acta2* [22, 23], endothelial cells (cluster 25–27) with *Cldn5*, *Pecam1*, *Cdh5*, and *Kdr* expression [24–26], and immune cells (cluster 28–31) with *Car2*, *Lcn2*, and *Cx3cr1* [27, 28] expression. The top differentially expressed genes (DEGs) in each cell type were shown in Fig. S1F, and mesenchymal and granulosa cells were accounted for most of the proportion (Fig. S1D).

### Identification and characterization of cell types and subpopulations

**Figure 2.**
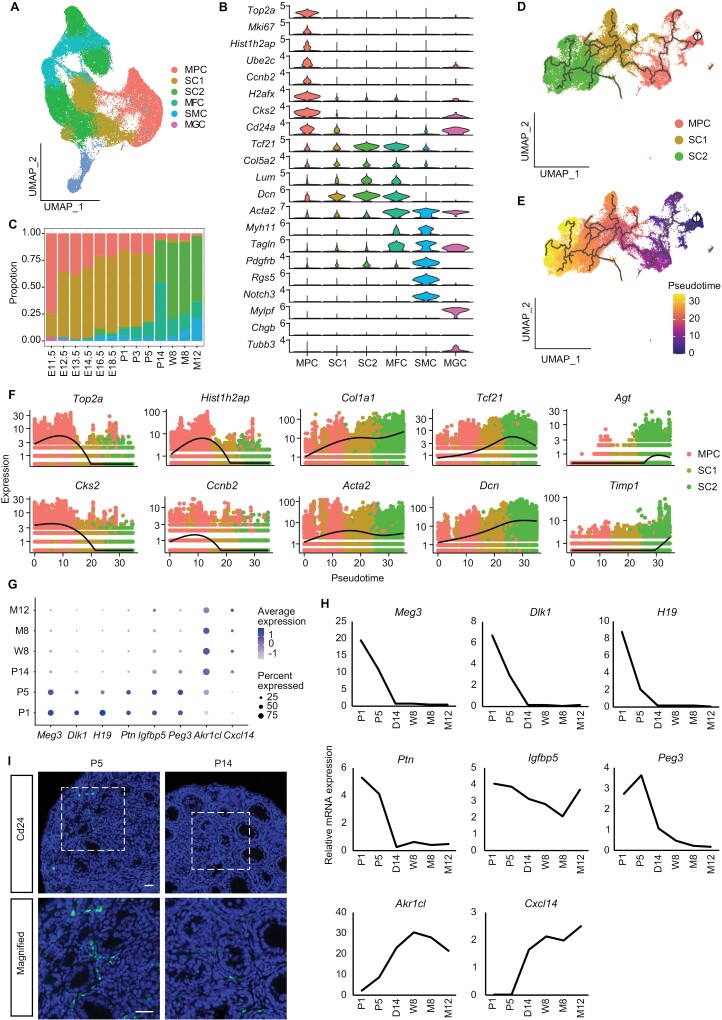
**Dynamics of mesenchymal cells during ovarian development.** (A) UMAP plot of mesenchymal cell distribution from E11.5 to M12, colored according to 6 identified cell subgroups: mesenchymal progenitor cell (MPC); type 1 stromal cell (SC1); type 2 stromal cell (SC2); myoblast fibroblasts cell (MFC); smooth muscle cell (SMC); myogenic cells (MGC). (B) Violin plot of marker gene expression in 6 mesenchymal cell subpopulations. (C) Cell number distributions of 6 mesenchymal clusters from E11.5 to M12. (D, E) UMAP visualization of cell trajectory from MPC to SC2. The developmental trace was colored by cell types (D) and inferred by pseudotime (E), respectively. (F) Expression pattern of selected marker genes along the pseudo-timeline which colored by cell type. (G) DotPlot of differentially expressed genes in the three types of stroma cells. (H) Relative expression of differentially expressed genes in the three types of stroma cells. (I) Immunofluorescence labeling of CD24 in P5 and P14 group. Bars = 50 μm.

To gain in-depth insight into ovary mesenchymal cells along the developmental line, we identified six distinct mesenchymal cell clusters according to the expression of canonical markers (Figs. 2A, 2B, S2A and S2B; Table S3), including myogenic cells (MGCs, *Mylpf*) [29], myofibroblasts (MFCs, *Acta2*, *Myh11*, and *Tagln*) [30], smooth muscle cells (SMCs, *Pdgfrb*, *Rgs5*, and *Notch3*) [31], and three stromal cell subpopulations according to their co-expressions of *Tcf21*, *Col5a2*, *Lum*, and *Dcn* [22]. These mesenchymal cell compositions also showed dynamics along the developmental line, for example, MGCs were exclusively identified in E11.5 gonads, and starting from E12.5, these cells were replaced by smooth muscle cells. Meanwhile, MFCs emerged at E16.5, coinciding with the onset of follicle formation (Fig. 2C). In the three subgroups of stromal cells, we named one cluster of cells as mesenchymal progenitor cells (MPCs) with highly expressed a cell surface stem cell marker CD24 and DNA replication related genes, such as *Top2a*, *Mki67*, and *Hist1h2ap* [11]. Immunostaining showed CD24^+^ cells scattered in the stroma of the P5 ovary; however, their numbers decreased significantly in the P14 ovary (Fig. 2I). This is consistent with the trend of cell proportion along the developmental timeline (Fig. 2C). The other two stromal cells, type 1 stromal cells (SC1s, *Meg3*, and *Igfbp5*) mainly existed in fetal and newborn ovaries (E11.5–P5), and type 2 stromal cells (SC2s, *Apoe* and *Cfh*) were found in P14–M12 ovaries (Figs. 2C and S2B). Next, to see the relationship among the three types of stromal cells, we performed pseudotime analysis and the result demonstrated the developmental line from MPCs to SC1 in fetal and newborn ovaries and then continued to differentiate into SC2s at P14 until ovarian senescence (Figs. 2D, 2E and S2C). GO and KEGG (Fig. S2D–F) analyses of pseudotime-associated genes further revealed the enrichment of DNA replication, cell division, or cell cycle-related terms in MPCs which is consistent with the high proliferate property of these cells, whereas the process of “cell adhesion” was highly enriched in both SC1 and SC2 stromal cells, suggesting that the two cell subpopulations may have active intercellular interactions with other cells. In addition, immunological and aging-related biological processes, such as “positive regulation of angiogenesis,” “immune system processes,” and “aging,” were specifically enriched in SC2 stromal cells. Some representative genes were plotted along pseudo-temporal trajectories, for example, the highly expressed cell division markers *Hist1h2ap*, *Top2a*, *Ccnb2*, and *Cks2* in MPCs and the abruptly elevated expression of ovarian senescence-associated genes *Agt* and *Timp1* in SC2s (Fig. 2F). The dynamics of some DEGs along the pseudo timeline were shown by RT-PCR of ovaries at different ages (Fig. 2G and 2H).

We also comprehensively explored cell subpopulations within the other five ovarian populations. Germ cells were identified from E11.5–P5 gonads or ovaries and divided into 8 subpopulations as previously reported (Fig. S3; Table S4), including pre-meiotic (*Hist1h2ap*), pre-leptoene (*Stra8*), leptoene (*Rec8*), zygotene (*Spo11*, *Meiob*, and *Rad51ap2*), pachytene (*Msh4*, *Mlh3*, and *Ybx2*), diplotene, dictyate, and dying germ cells [9]. Granulosa cells were classified into pre-granulosa (E11.5–P5) and granulosa cells (P5–M12). According to previous studies, the pre-granulosa cells were further divided into bipotential cells (BPC, *Wnt4*, and *Wnt6*), bipotential pre-granulosa cells (BPG, *Foxl2*, and *Hmgcs2*), and epithelial pre-granulosa cells (EPG, *Lgr5*, and *Gng13*) [32, 33]. Starting from P5, both BPG and EPG were gradually replaced by granulosa cells (*Foxl2*, *Amh*, and *Htra1*) and accompanied by follicular development to the antral stage, granulosa cells differentiated into cumulus cells (*Inhba*, *Hspa5*) [34] and after ovulation, they changed to luteum cells (*Star* and *Lhcgr*) (Fig. S4; Table S5) [35]. Theca cells were divided into three subpopulations (Fig. S5A–E; Table S6), including theca progenitor cells (TPCs, *Wt1* and *Ptch1*) [36, 37], theca cell 1 (TC1s, *Cyp17a1*) [21], and theca cell 2 (TC2s, *Star* and *Lhcgr*) [38]. Epithelial cells were classified into four clusters, including epithelial stem cells (ESCs, *Cdk1*, *Ccna2*, and *Cenpa*) [11], type 1 epithelial cells (EC1s, *Itm2a* and *Ptn*), type 2 epithelial cells (EC2s, *Tnni1*), and type 3 epithelial cells (EC3, *Ly6e*) (Fig. S5F–J; Table S7). EC1s were mainly presented in an undifferentiated gonad (E11.5 and E12.5). EC2s were found in fetal and newborn ovaries from E13.5 until P5 and after that, they were replaced with dominant EC3s. Immune cells were classified into 9 subpopulations (Fig. S6; Table S8), including macrophage (*Cd68* and *Cd14*) [39], monocytes (*S100a4* and *Ccr2*), dendritic cells (*Cd209a*) [40], neutrophils (*Pglyrp1* and *Lcn2*), granulocytes (*S100a8*), T cells (*Cd3d* and *Cd3g*), B cells (*Cd79b* and *Mzb1*), erythoroid cells (*Cpox* and *Car2*), and basophils (*Cpa3* and *Cd63*). Thus, from a global analysis of single cells from ovaries at different developmental stages, cell types and the dynamics of subpopulations in each cell type were well depicted along the developmental timeline.

### Overview of cell–cell interactions in mouse ovary across development

**Figure 3.**
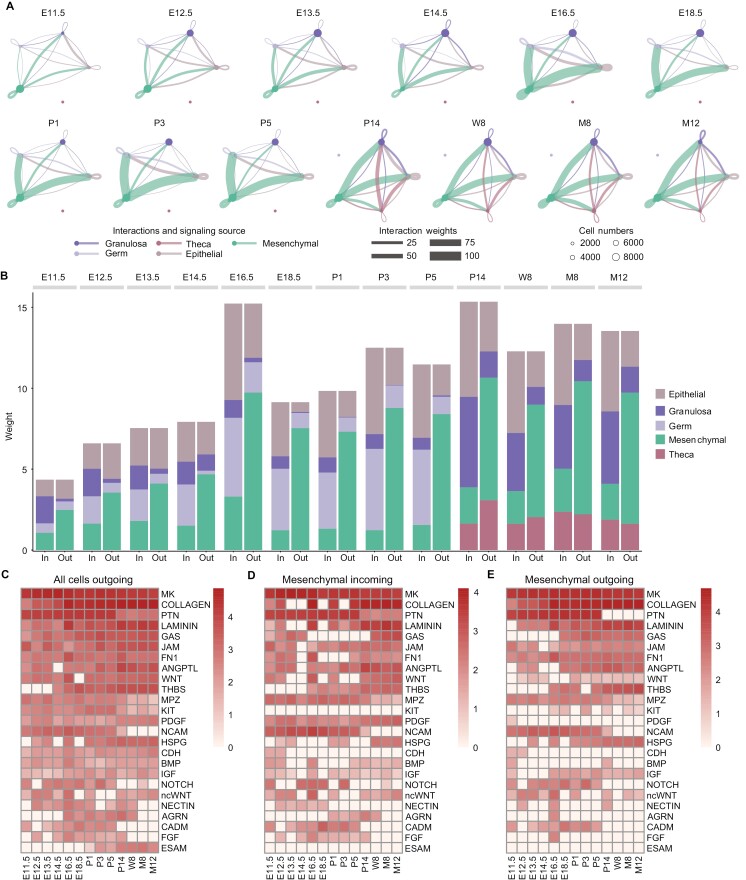
**Landscape of intercellular communications in mouse ovary from E11.5 to M12.** (A) The strength of intercellular communications among major cell types in the ovary at different developmental stages (E11.5–M12). Circle sizes, edge width, and edge color represent cell numbers, communication strength, and signaling source, respectively. (B) Stacked bar plot of the aggregated incoming and outgoing signaling strength of each cell population from E11.5–M12. The bar color is the same with the edge color in (A). (C) Heatmap of the outgoing signaling strength of the top 25 signaling pathways derived from the intercellular communications among major cell types in mouse ovary from E11.5–M12. (D, E) Heatmap of the incoming (D) and outgoing (E) signaling strength of the top 25 signaling pathways derived from the intercellular communications of mesenchymal cells in mouse ovary from E11.5–M12.

We then used CellChat, a widely utilized tool for analyzing cell–cell communication, to investigate crosstalk between diverse cell types at each developmental time point (E11.5–M12, Fig. 3A) [41]. Overall, the cell–cell communications increased gradually during E11.5–P5 when the ovary underwent tissue construction, stabilized at a high level at P14 when the ovarian structure was well developed, and decreased after M8 as the ovary was aged (Fig. 3B). Among cell types, mesenchymal cells played a major role in outgoing signals, accounting for over 50% of communications at each time point. The signal strength dramatically increased in E16.5 when cyst breakdown and follicle assembly started, and thereafter, it remained the strongest among outgoing signals until M12 in the aged ovary. In contrast, the incoming signals of each cell type in the ovary were relatively balanced. Another transition point occurs at P14 when the theca layer forms around the growing secondary follicles. At this stage, the communication strength to granulosa cells increases significantly, and the theca cells begin to participate in intercellular communication by integrating both incoming and outgoing signals (Fig. 3B).

Totally, 388 ligand–receptor pairs were identified for cell–cell communication between different cell types, which can be categorized into 56 important signaling pathways. Among the top 25 outgoing pathways (Fig. 3C), the Midkine (MK) signaling pathway emerged as the strongest from E11.5 to M12. This pathway was also identified as the most prominent pathway when analyzing the signal strength specifically from mesenchymal cells (Fig. 3D and 3E). In contrast to MK, pleiotrophin (PTN) signals from mesenchymal cells decreased significantly in adult ovaries. MK and PTN belong to the heparin-binding growth factor family, regulating cell growth, survival, migration, differentiation, and angiogenesis. Deletion of either *Mdk* or *Ptn* alone does not disrupt non-neurological tissue development, but double deficiency leads to infertility in female mice [42, 43]. Thus, MK and PTN may function differently at different stages of ovarian development. Growth arrest-specific (GAS) signals were observed emerging from mesenchymal cells starting at E18.5 and persisting until M12. Given that *Gas2* mutant females exhibit fertility issues and disrupted folliculogenesis, the mesenchymal GAS signals likely play a crucial role in follicular development [44].

### Dynamics of cell–cell communication among somatic subpopulations in mouse ovary

**Figure 4.**
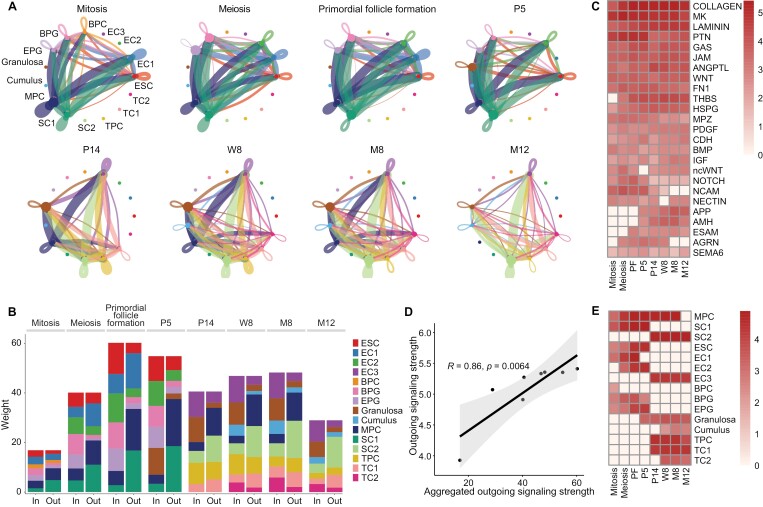
**Dynamics of intercellular communication among somatic cells in the mouse ovary.** (A) The strength of intercellular communications among somatic subpopulations. The ovarian developmental stages were characterized as mitotic stage (mitosis, E11.5–E12.5), meiotic stage (E13.5–E16.5), primordial follicle formation (E18.5–P3), and P5, P14, W8, M8, and M12. Circle sizes, edge width, and edge color represent cell numbers, communication strength, and signaling source, respectively. (B) Stacked bar plot of the aggregated incoming and outgoing signaling strength of each somatic subpopulation during mouse ovary development stages. The bar color is the same as the edge color in (A). (C) Heatmap of the outgoing strength of the top 25 signaling pathways derived from the intercellular communications in somatic subpopulations at different development stages. (D) The scatter plot of the outgoing communication strength of collagen pathway and aggregated outgoing communication strength in somatic subpopulations during ovarian development. (E) Heatmap of the outgoing strength of collagen signaling in somatic subpopulations at different stages of ovarian development.

To delve deeper into the influence of intercellular communications on ovarian development, we segmented ovarian cells into eight key stages: mitosis (E11.5–E12.5) [45], meiosis (E13.5–E16.5) [45], primordial follicle formation (E18.5–P3) [45], P5 (primordial follicle stage), P14 (prepuberty, follicle growth stage), W8 (young), M8 (fertility decreased stage), and M12 (sterility, aged stage). Cell–cell communications of 15 subpopulations of somatic cells (ESC, EC1–3 epithelial cells; BPC, BPG, and EPG pre-granulosa cells; granulosa cells and cumulus cells; MPC, SC1, and SC2 stromal cells; TPC, TC1, and TC2 theca cells) were explored across these stages (Fig. 4A and 4B). During primordial follicle formation, MPCs, SC1, and EC1 emerged as the most active communicators. Notably, while the proportion of MPCs declined over development, their signal strength remained high until ovarian senescence, implicating their vital role in maintaining ovarian structure and function. EC1 epithelial cells, distinct from ESC and EC2, primarily transmitted signals during follicle formation, suggesting their collaboration with MPCs and SC1 in directing cellular differentiation during the process. Throughout ovarian development, granulosa cells primarily functioned as signal receivers, whereas TPCs interacted bidirectionally, both sending and receiving signals. When focusing solely on somatic cells, collagen signaling was identified as the most enriched pathway, strongly correlated with overall signaling strength (*r* = 0.86, *P* < 0.001) (Fig. 4C and 4D). The contribution of each somatic cell group on the signaling pathway and the dynamics of outgoing signal strength is shown in Fig. 4E. Therefore, the analysis highlights the pivotal role of collagen signaling in guiding somatic cellular communications in the ovary across all developmental stages.

### Collagen signaling pathway elaborates the role of stromal cells in cell–cell communications in the mouse ovary

To comprehend the dynamics and the impact of collagen signaling on ovarian development, we mapped the intercellular collagen communication network and analyzed its centrality (Figs. S7A and 5A). The heatmaps revealed the evolving roles of cell groups: MPCs transition from receivers/mediators to senders and function as influencers alongside SC1 during follicle formation. Subsequently, SC1 was replaced with SC2, and until ovarian aging, MPCs kept on actively communicating with other somatic cells. Epithelial subgroups also shift roles, with EC1 dominating as a sender during primordial follicle formation, while ESC and EC2 adapt to mediate and receive signals. Granulosa cells consistently receive and influence collagen signals. Theca subgroups exhibit diverse roles, with TPC mediating at P14 and 8W, TC2 receiving signals, and TC1 gradually becoming an influencer.

**Figure 5.**
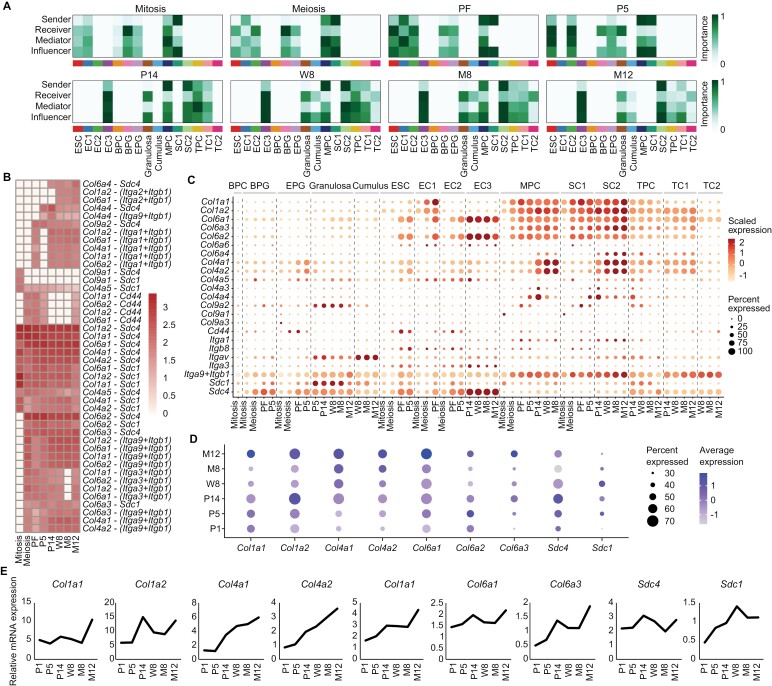
**Identification of signaling roles of somatic subpopulations in the collagen signaling pathway.** (A) Heatmap of the relative importance of signaling roles of each somatic in collagen signaling network at mouse ovary development stages. (B) Heatmap of the contribution of each ligand–receptor pair to collagen signaling pathway. (C) Expression distribution of collagen signaling-related ligand–receptor genes in epithelial, granulosa, stromal, and theca subpopulations at each development stage. (D) Expression of collagen-related genes in mouse ovaries of several development stages. (E) RT–PCR showed relative expression of collagen-related genes in mouse ovaries of several development stages.

The ligand–receptor pairs of the collagen signaling network were then investigated and the top 25 ligand–receptor pairs were shown in Fig. 5B. In addition, we also demonstrated the most important ligand–receptor pairs of the collagen signaling sending from stromal cells to stromal cells (Fig. S7B), to granulosa cells (Fig. S7C), to epithelial cells (Fig. S7D), and to theca cells (Fig. S7E). Gene expressions of these pairs were cell-type and developmental stage-specific (Fig. 5C). For instance, *Col1a1* and *Col1a2* are ubiquitous in stromal cells, whereas *Col4a1* and *Col4a2* increase in MPCs and SC2 post-W8. EC3 uniquely exhibits high levels of *Col6a1* and *Col6a2* and the integrin-coding gene *Itgav* shows specific expression in cumulus cells. The expression of syndecan family members, *Sdc1*, increases in BPG/EPG and keeps high levels in adult granulosa cells but decreases as ovaries age, while *Sdc4* remains consistently elevated in EC3. RT–PCR confirmed the dynamic expression of collagen family members and receptors across development (Fig. 5D and 5E).

### The influence of cell–cell communication on ovary aging

**Figure 6.**
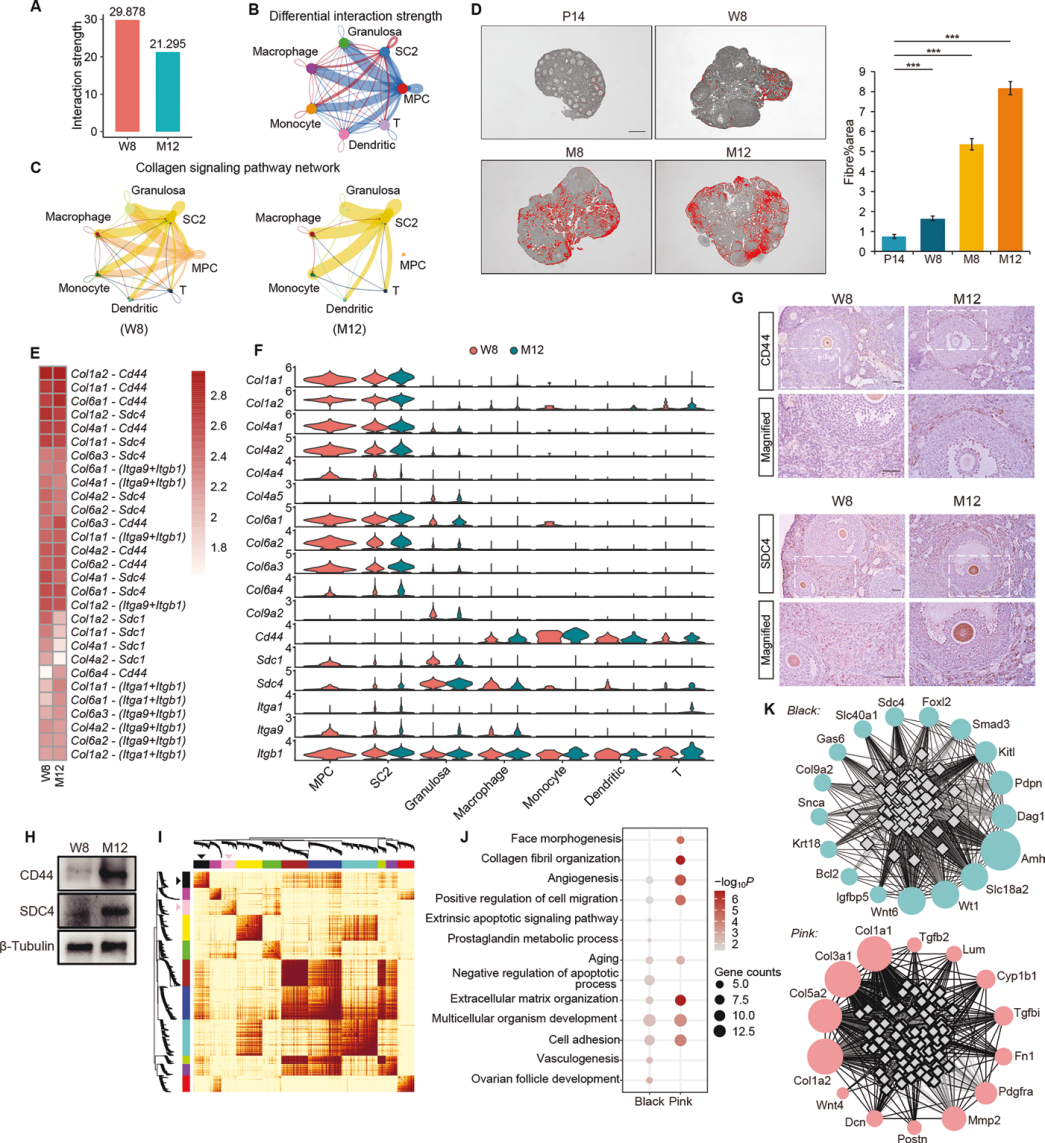
**The involvement of collagen signaling pathway in the process of ovarian aging.** Intercellular communications among stromal cells (MPCs and SC2 stromal cells), granulosa cells, and immune cells (macrophage, monocyte, dendritic cells, and T cells) were analyzed for comparison between W8 and M12 ovaries. (A) Comparison of the total interaction strength among somatic cell types. (B) Circle plot of differential interaction strength among somatic cell types. Edges represent increased and decreased interaction strength in M12 (right) as compared to W8 (left) ovaries. (C) Circle plot of the communication network of the collagen signaling pathway in W8 and M12 ovaries. Circle size, edge width, and edge color represent cell numbers, communication strength, and signaling source, respectively. (D) Representative ovarian sections with Masson staining of collagen and fibrotic quantification in ovaries from P14 to 12M of age. Bar = 100 μm. Fibrotic area (mean ± SEM) was shown as relative to stromal tissue area. ***P < 0.0001. (E) The heatmap showed the contribution of each ligand–receptor pair to collagen signaling pathway between W8 and M12 ovaries. (F) Violin plots of collagen signaling-related genes between W8 and M12 ovaries. (G) Immunostaining of CD44 and SDC4 in ovaries collected from 8W and 12M groups. Bars = 50 μm. (H) Western blot of CD44 and SDC4 expression in 8W and 12M group. (I) The heatmap depicts the Topological Overlap Matrix (TOM) of genes selected for weighted co-expression network analysis. Light color represents lower overlap and red represents higher overlap. The black triangle and pink triangle refer to the selected modules with enriched genes for GO and KEGG analysis. (J) GO enrichment analysis of genes in black and pink modules. (K) Co-expression network of genes in pink (top) and black (bottom) modules.

Our previous study has demonstrated the association of altered macrophage states with local pro-inflammation environments in aged ovaries [46]. To further explore the potential role of stromal cells during ovarian aging, we compared the intercellular communication networks established by stromal subgroups, immune subgroups, and granulosa cells between W8 and M12 ovaries. Overall, the strength of intercellular communications decreased in M12 ovary and accompanied with the depletion of MPCs, SC2 stromal cells showed increased interactions with other cells (Fig. 6A and 6B). The strength of collagen pathway from MPCs and SC2 also showed a similar trend between 8W and 12M ovaries (Fig. 6C). Consistent with previous reports, the deposition of collagen fibers in the ovarian stroma was then manifested by Masson staining during the aging process (Fig. 6D) [47]. Receptor–ligand analysis identified CD44 and SDC4 as the dominant receptor which contributed mostly to the collagen signaling network between 8W and 12M ovaries (Fig. 6E). The expression of enriched ligand and receptor genes of the collagen signaling pathway was shown in Fig. 6F and the communication probabilities from MPCs or SC2 stromal cells to various immune cell types and other somatic cells that mediated by each receptor–ligand pair was shown in Fig. S8A and S8B. Immunostaining showed the expression of CD44 and SDC4 in the stroma of 8W and 12M ovary (Fig. 6G) and increased protein levels were observed in 12M ovary (Fig. 6H). CD44 was reported to be involved in inflammatory response, and was part of the macrophage migration inhibitor receptor complex [48]. The results indicated CD44- or SDC4-mediated collagen signals may play a vital role in cellular communications during ovarian aging.

To further understand how cell–cell communications influence mouse ovary aging, we performed weighted gene co-expression network analysis (WGCNA) [49] of highly variable genes identified in granulosa cells and stromal cells in W8 and M12 ovaries. In total, 1451 genes were included to generate the co-expression networks, and 11 functional modules were identified and labeled as different colors (Fig. 6I; Table S9–S11). GO analysis showed that gene functions in the pink module were related to collagen fibril organization, extracellular matrix organization, cell migration, and cell adhesion, while genes in the black module were enriched in terms related to aging, cell apoptosis, and ovarian follicle development (Fig. 6J and 6K; Table S10). The results suggest the pivotal role of stromal cells, especially MPCs in regulating the ovarian immune-microenvironment during ovarian aging.

### Activation of MPCs is enough for stroma remodeling in old ovaries

**Figure 7.**
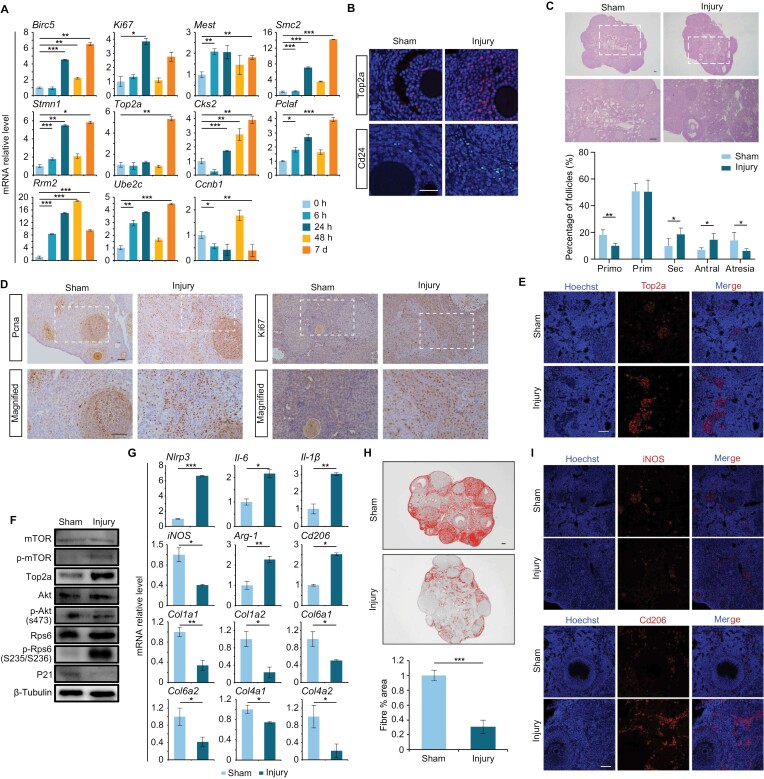
**Activation of MPCs after ovarian injury and its roles in tissue remodeling during ovarian aging.** 8W or 10M female mice were used for ovarian partial resection, with one lateral ovary for operation, and the other side as sham control. Ovaries were collected at 0 h, 6 h, 24 h, 48 h, 5 day, or 7 day in 8W operated mice and at 21 days in 10M mice. (A) Relative mRNA levels of MPCs marker genes detected by RT–PCR. Data are presented as means SEM of at least three replicates. The levels of all tested mRNAs in 0 h group were set to 1. *P < 0.05, **P < 0.01, ***P < 0.001, compared with the 0 h group. (B) Immunofluorescence of TOP2A and CD24 in sham and injury group. Bar = 50 μm. (C) Ovarian histology and follicle counting between paired ovaries. White frame: the image was magnified in the lower panel. Lower figure: *n* = 4 pairs of ovaries for follicle counting. **P < 0.01. Bars = 100 μm. (D) Immunostaining of PCNA and Ki67 in paired ovaries collected from 10M mice. White frame: the image was magnified in the lower panel. Bars = 50 μm. (E) Immunofluorescence of TOP2A in sham and injury group of 10M mice. (F) Western blot of p-mTOR, p-AKT, p-RPS6, TOP2A, and P21 expressions in sham and injury group of 10M mice. The expressions of mTOR, AKT, RPS6, and β-Tubulin were used as internal controls. (G) Relative expression of inflammation-related genes and collagen family members in sham and injured group of 10M mice. Data are presented as means ± SEM of at least three replicates. The levels of all tested mRNAs in the sham group were set to 1, *P < 0.05; **P < 0.01, ***P < 0.001, compared with the sham group. (H) Representative processed color threshold images of Masson-stained ovarian tissue sections between sham and injured ovaries at 10M age. Lower panel: Quantification of ovarian fibrosis in sham and injured ovaries. *n* = 4 pairs ovaries. ***P < 0.001. Bar = 100 μm. (I) Immunofluorescence of iNOS and CD206 in sham and injured ovaries of 10M mice. iNOS and CD206 represent different polarized macrophage states, M1 and M2 macrophage, respectively. Bar = 50 μm.

As the origin of ovarian stroma cells, our study has revealed the central role of MPCs in intercellular communications with other cell types. Our previous study has demonstrated the change of ovarian microenvironment after ovarian injury [46]. Based on the potential role of MPCs on the remodeling of ovarian stromal cells, we suppose it may function during the process. Because MPCs were hardly detected in 12M ovary, we next to see if the ovarian injury can activate MPCs and alter the ovarian microenvironment in 8W and 10M ovaries. Ovarian surgery was performed with partial section on one lateral ovary and the other side served as sham control. In 8W mice, we observed time-dependent increase of some representative genes of MPCs in injured ovaries, and after 5 days of surgery, immunostaining demonstrated the increased Top2a and CD24 positive cells in ovarian stroma (Fig.7A and 7B). In 10M mice, ovarian morphology showed growing follicles in injured ovary after 21 days of surgery. Follicle counting revealed a significantly decreased percentage of primordial follicles and follicle atresia, while an increase in secondary and antral follicles was observed (Fig. 7C). The increase of PCNA/Ki67/TOP2A-positive cells was also observed in the stroma of injured ovary (Fig. 7D and 7E). Similar to what we observed in the previous study, ovarian injury also induced the activation of the mTOR signaling pathway with increased phosphorylation of mTOR and RPS6. Moreover, we detected the significant increase in TOP2A protein levels and the decrease in P21 expression, an age-associated cell cycle-related protein after surgery (Fig. 7F). RT–PCR showed the differential expression of pro- and anti-inflammatory genes between bilateral ovaries, such as *Il-6*, *Il-1β*, *Nlrp3*, *iNOS*, *Arg-1,* or *Cd206*. The altered expression also included collagen ligands *Col1a1*, *Col1a2*, *Col6a1*, *Col6a2*, *Col4a1,* and *Col4a2* (Fig. 7G). Consistent with the RT–PCR result, we found the decreased fibrosis in the injured ovary of 10M old mice (Fig. 7H). Immunostaining further illuminated the transition of M1 and M2 macrophages with increased expression of M2 macrophage-related CD206 in the injured ovarian stroma (Fig. 7I). Taken together, our results reveal the activation of MPCs and its role in remodeling ovarian microenvironment after ovarian injury.

## Discussion

**Figure 8.**
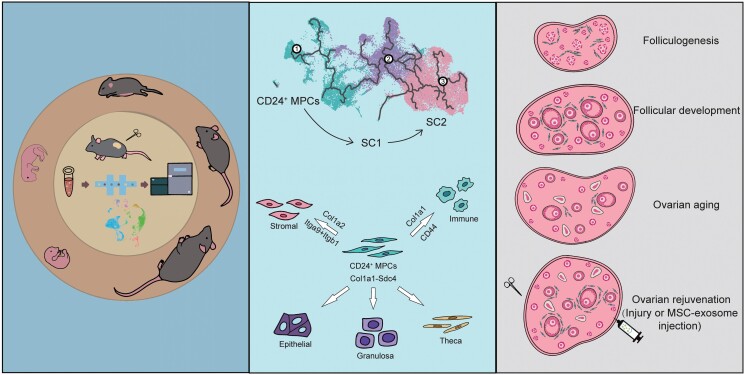
**Graphical summary of the current study.**The figure was created using materials from the SciDraw website.

With the development of the single-cell sequencing technique, it is possible for us to delineate the dynamic gene expressions and intercellular communications among various cell types in the ovary at a special developmental stage. However, it still lacks an overall understanding about how ovarian cells communicate with each other in directing ovarian development from the fetal to adult stage until senescence. In this study, we integrated 106,579 mouse ovary cells from E11.5 to M12 of age in view of gene expression and intercellular communications. The cell types were well classified and the developmental trajectories of each cell type, including stromal cells, germ cells, epithelia cells, granulosa cells, and theca cells, were accurately depicted. We identified CD24^+^ MPCs as the progenitor cell type guiding stroma differentiation from SC1 to SC2 stroma cells during the establishment of ovarian structure, particularly when primordial follicle form. In addition, MPCs function as key regulators, influencing interactions with other cell types through specific ligand–receptor pairs of the collagen signaling pathway. As the ovary ages, there is a notable decrease in the number of MPCs. Nevertheless, the ovary can experience rejuvenation through the activation of MPCs, either induced by ovarian injury or achieved via mesenchymal stem cell (MSCs) transplantation (Fig. 8). This comprehensive analysis not only enhances our understanding of the cellular dynamics underlying ovarian development but also provides insights into potential therapeutic strategies for age-related ovarian decline.

In recent years, the heterogeneity of mesenchymal/stromal cells has become more evident due to the advancements in lineage-tracing methodologies and single-cell transcriptome analysis. This heterogeneity is observed not only across different tissues or within a specific tissue under various physiological and pathological conditions but also based on the relative location of the cells within the tissue microenvironment and their interactions with other cell types [50]. Ovarian mesenchymal cells also demonstrate such heterogeneity during the whole lifetime. For example, neuron cells or myogenic progenitor cells are limited in fetal gonad as early as E11.5 and E12.5 with very few cell numbers. We then separated MFC myofibroblast and SMC smooth muscle cells from stroma cells and identified MPCs as the origin of two kinds of stroma cells, SC1 and SC2. The two stroma cell types reflected the change of stroma cell properties after P5 when ovarian structure was established. When comparing the differential gene expression between SC1 and SC2 stromal cells, the enriched genes in SC2 are related to angiogenesis, aging, immune response, and ECM organization. This is consistent with the complex role of SC2 stromal cells as signal senders in communicating with other cell types to maintain ovarian microenvironment, direct follicular development, or induce ovarian aging in adult ovary. Previous studies with cyclic adult ovaries have demonstrated two interstitial stromal cell types, steroidogenic stromal cells, and fibroblast-like cells [51]; a bioRxiv preprint version of the study divided stromal cells as stromal progenitor cell, structure stromal cell, perifollicular stromal cell and steroidogenic stromal cell [52]. After careful comparison, structure stromal cells referred to SC1 and SC2 stromal cells, whereas perifollicular stromal cells and steroidogenic stromal cells represented TC1 and TC2 theca cells in our study. In the study, we also identified TPC theca progenitor cells with significant enrichment of *Wt1*, *Ptch1,* and *Bgn* as markers. According to the differential gene expressions, that is consistent with the reported human theca progenitor cell [53]. Thus, our results manifest the point that theca origins at least in part from Wt1^+^ cells in stroma [36, 54]. Moreover, we got similar but more detailed mesenchymal cell types and developmental traces from gonad development until ovarian aging.

In the ovary, oocytes develop in follicles embedded in a complex ovarian stroma which consists of various cell types and ECM. ECM which is composed of collagens, elastin, glycoproteins, and proteoglycans, not only provides physical support for a tissue but also stimulates signaling pathways in cells to affect tissue morphogenesis, differentiation, and homeostatic status [6]. Critical alterations in ECM composition and organization have often been reported to impact tissue function. For example, excessive deposition of ECM, characterized by collagen I and II, induces fibrosis in several aging tissues, including the heart, lung, and liver [55]. Fibrosis in the aged ovary was also related to reduced oocyte quantity and quality. Inhibition of fibrosis by antifibrosis drugs could restore ovarian function and delay ovarian aging in old mice [47]. However, how ECM property modifications regulate cell-to-matrix interactions and direct gonad and ovarian development until senescence lacks a comprehensive view. In the study, with the advancement of single-cell sequencing technology, the intercellular communications of somatic cells and the participated signaling pathways have been well documented along the developmental line. As the most dominant ECM component, the collagen signaling pathway was identified as the major outgoing signals in stromal cells to communicate with other somatic cell types, especially with granulosa and epithelial cells. Ligand–receptor pairs analysis then revealed the dynamic changes of collagen ligands and receptors in various somatic cell types at different development stages. This is the first time that the intercellular signal transductions between somatic cells have been well depicted in the whole life of the ovary. It has been demonstrated that the imbalance between M1 and M2 macrophages contributes to the increased expression of inflammatory cytokines and collagen deposition during ovarian aging [56]. Based on the association of a pro-inflammatory microenvironment with ovarian aging, we involved immune cells to compare the alterations of cellular communications between 8W and 12M ovaries. We observed strengthened outgoing collagen signals from SC2 stromal cells to macrophage and monocyte in 12M ovary and enriched specific ligand–receptor pairs that may function in the aging process. The deposition of collagen and increased expression of collagen receptors CD44 and SDC4 in the stroma were further verified in the old ovary. Together, our results not only revealed the major role of the stromal collagen signaling pathway in ovarian development and ovarian aging but also identified a series of stage-specific ligand–receptor pairs that may function during the process.

Fibroblasts are highly dynamic cells that play an essential role in maintaining the structure integrity of virtually each organ through producing and remodeling ECM [47]. In recent years, the fibroblast heterogeneity has been well understood with significant phenotypic and functional variability even within the same tissue. Such heterogeneity is also reflected at stages of tissue development and from alterations in the tissue microenvironment [55, 57]. In the study, we demonstrated the heterogeneity of fibroblasts and identified MPCs as the origin to direct the differentiation of SC1 and SC2 stromal cells during ovarian development. The MPCs highly expressed cell division genes which the property is similar to mesenchymal progenitors reported in normal and fibrotic lung tissue [30]. However, different from the study in lung, we found MPCs in the ovary specifically expressed stem cell marker CD24 [58]. CD24^+^ progenitors have been shown to repair kidney injury and reverse renal fibrosis in experimental animals and even in humans [59]. CD24-deficient mice also showed delayed wound healing and impaired bone healing at tooth extraction sites [60, 61]. The CD24^+^ MPCs in the ovary have similar stem cell property, for example, although its numbers decreased significantly in adult ovary, the cells kept highly intercellular communication strength with other somatic cell types until ovarian aging when they were hardly detected. Our study revealed that MPCs could be activated after ovarian injury or following intra-bursal injection of exosomes derived from human umbilical mesenchymal stem cells (Fig. S8C) [62]. The results demonstrated the advantages of this approach in promoting tissue repair and alleviating stromal fibrosis in the aged ovary. Thus, we identified ovarian CD24^+^ MPCs as the major player in directing stroma cell differentiation and conducting intercellular communications with other somatic cell types during both fetal and adult life. Significant decrease or depletion of the cells in the ovary may lead to deteriorated follicular development and stroma fibrosis. Although it is disputed about the female germline stem cell and its potential for regeneration of the oocyte pool in adulthood, multipotent mesenchymal stem cells have been identified in mouse and human adult ovaries with the ability to differentiate into other cell types such as adipocytes, chondrocytes, or osteocytes [63, 64]. Future studies will focus on the stem cell property of MPCs and the underlying mechanism of how it regulates stromal cell differentiation and senescence.

## Research limitations

In this study, we explored the dynamics of ovarian stroma across the entire lifespan of the mouse ovary. We also illuminated the pivotal role of CD24^+^ MPCs in maintaining homeostasis during ovarian development. Although MPCs in aged ovaries can be activated by ovarian injury or following ovarian injection with MSCs, the properties of these cells remain unclear. Specifically, are MPCs a type of mesenchymal stem cells unique to the ovary? How do they orchestrate somatic cell differentiation at different stages during ovarian development, and what is the relationship between their activity and ovarian aging? Since MPCs keep very low numbers in adult ovaries, can these cells be propagated *in vitro*, serving as a reservoir to restore declining ovarian functions? These are questions we seek to explore further.

## Method

### Research ethics

This study was approved by the Ethics Committee of Nanjing Medical University (code number:1811050-1). All animal procedures were approved by the Animal Experimental Center of Nanjing Medical University.

### Experimental animals

C57BL/6J strain female mice at different developmental stages (P1, P14, W8, M8, M12) were purchased from Vital River Laboratory Animal Technology Co. (Beijing, China) for single-cell RNA sequencing. To see the effect of mesenchymal progenitor cells on ovarian stroma remodeling, partial ovarian resection was performed according to the previous study [65]. Briefly, mice at 8W or 10M were chosen with partial ovarian resection on the lateral ovary and the other lateral ovary left unoperated as a control. For 8W mice, ovaries were collected at different timepoints (0 h, 6 h, 24 h, 48 h, D5, and D7) to evaluate the activation of MPC. For 10M mice, ovaries were collected after 3W of surgery. All mice were housed in the Animal Core Facility of Nanjing Medical University (Nanjing, China). Mice were maintained under a 12 h dark/12 h light cycle at 22°C with free access to food and water. The experimental protocol was approved by the Committee on the Ethics of Animal Experiments of Nanjing Medical University.

### Single-cell preparation and sequencing

Ovaries were dissected and digested in 500 µL HBSS supplemented with 0.25% trypsin, 1 mM ethylene diamine tetraacetic acid (EDTA), and 0.01% DNase I at 37°C for 10 min with gentle agitation. To stop the digestion, 500 µL HBSS (plus 10% FBS) was added and the cell suspensions were centrifuged at 400 *g* for 5 min at 4°C. After aspirating the supernatant completely, the cells were resuspended in 500 µL HBSS. The dissociated single-cell suspensions were filtered through 40 μm cell strainers (Greenpia Technology, 2-200203, Korea) and washed 2 times with PBS containing 0.04% BSA. Cell viability was evaluated by staining in 0.4% Trypan Blue and available cell concentrations (1000 cells/μL) were prepared for single-cell sequencing by a 10x Genomics Chromium system. The fragment length distribution and the effective concentration of the library were evaluated by Agilent 2100 High Sensitivity DNA Assay Kit (Agilent Technologies, CA, USA) and Qubit 3.0 Flurometer (Life Technologies, CA, USA). The effective concentration of the library aimed > 10 nmol/L and pair-end 150 bp sequencing was performed to produce high-quality data on Illumina novaseq 6000.

### scRNA-seq data analysis and cell type annotation

Single-cell sequencing datasets were analyzed by package CellRanger v3.1.0 and Seurat v4.0.5 [66]. The 10x Genomics pre-built mouse genome for mm10-3.0.0 was used as a reference. High-quality quality control processes and standard batch effect removal methods to ensure the consistency of the data were utilized. Throughout the data preprocessing, normalization, dimensionality reduction, batch correction, and subsequent analyses, (i) we normalized the expression levels to eliminate technical biases, (ii) removed low-quality cells that those with excessively low or high expression levels, (iii) eliminated genes with low expression or that constitute background noise, and (iv) restricted cells based on the number of detected features, count values, the proportion of red blood cell genes, and the proportion of mitochondrial genes. In detail, “NormalizeData” function in Seurat v4.0.5 was used for normalization, and low-quality cells were removed using “subset” function. Putative doublets, cells with minimal genes less than 200 or genes expressed in less than 3 cells, and cells with a high proportion of mitochondrial genes or red blood cell genes were filtered by DoubletFinder (v2.0.3) [67] for downstream analysis. To integrate the datasets of mouse ovary from E11.5 to M12, we detected 2000 features with high variation using “FindVariableFeatures” function in Seurat v4.0.5, and then we identified ‘anchors’ between individual datasets with the module “FindIntegrationAnchors.” Finally, these anchors were passed to the “IntegrateData” function to correct batch effect. Cell clusters were characterized by “FindNeighbors” (dims = 1:20) and “FindClusters” (resolution = 0.6) and visualized with Seurat function “RunUMAP” based on the UMAP algorithm with dims = 1:20. Each cell cluster was classified and manually annotated based on the expression of canonical marker genes of particular cell types. We used the Seurat function “FindAllMarkers” to identify conserved markers and DEGs in each cell cluster with parameter log2(fold change) < 0.5 and FDR < 0.05. GO and KEGG enrichment analysis of the DEGs was conducted using the DAVID webtool [68] with FDR < 0.05.

### Cell trajectory analysis

Monocle3 [69] was used for single-cell trajectory analysis of subpopulations of mesenchymal, granulosa, germ, theca, and epithelial cells. With the gene count matrix of diverse cell subpopulations as input to create Monocle3 object, the function of “preprocess_cds” was used to normalize and preprocess the data, and “align_cds” was used to remove batch effects with cell alignment. To identify the trajectory of cell subpopulations, we first used the function “reduce_dimension” to reduce the dimensions, and “cluster_cells” to cluster cells. Then, function “learn_graph” was used to learn the trajectory graph, and “order_cells” was used to order the cells according to their progress through the developmental program. In addition, we also use the function “graph_test” to identify genes that change as cells progress along a trajectory and “find_gene_modules” to collect the trajectory-variable genes into modules.

### Inference and analysis of cell–cell communication by CellChat

Cell–cell interactions based on the expression of known ligand–receptor pairs across diverse cell types were analyzed by CellChat3.2 (v.0.02) [41]. We followed the CellChat official workflow to infer potential cell–cell communication networks induced in mouse ovary from E11.5 to M12. The normalized counts of gene expression across cell types derived from Seurat were served as input for CellChat and pre-processed using functions identifyOverExpressedGenes, identifyOverExpressedInteractions, and projectData with default parameters. We used the mouse-secreted signaling, ECM-receptor, and cell–cell communication database as a priori network information. Cell–cell communication networks were then established by functions “computeCommunProb” to compute the communication probability and infer cellular communication network, “computeCommunProbPathway” to infer the cell–cell communication at a signaling pathway level, and “aggregateNet” to calculate the aggregated cell–cell communication network with default parameters and fixed randomization seeds. Communication strengths of each signaling pathway at different time points were visualized by function “pheatmap”. The key signal pathways and the contribution of each ligand–receptor pair to the signaling pathway were analyzed by functions “netVisual_aggregate” and “netAnalysis_contribution.” “netAnalysis_computeCentrality” and “netAnalysis_signalingRole_network” were used to compute, visualize the network centrality scores, and identify signaling roles (dominant senders, receivers, influencers and mediators) of cell groups. “plotGeneExpression” was used to plot the signaling gene expressions. To compare the interaction among different cell populations at different development stages, we used “compareInteractions” to compare the total interaction strength, “netVisual_diffInteraction” to compare the differential number of interactions or interaction strength among different cell populations.

### Gene co-expression network analysis

WGCNA [49] was applied to explore gene co-expression pattern in granulosa cells and macrophages. First, we evaluated 1451 variable genes in these two cell types and generate the gene–cell expression matrix as input. After topology analysis of our data used by WGNCA (1.70-3) R packages, we chose 3 as the soft threshold for subsequent analysis. The co-expression network and corresponding gene modules were generated by the blockwiseModules function with parameters “mergeCutHeight=0.25 and minModuleSize=50.” GO analysis of different modules was carried out by David (6.8) with *P*-value < 0.05. Module gene co-expression networks were visualized using Cytoscape(3.7.2).

### Immunohistochemistry and immunofluorescence

Samples were fixed in 10% formalin overnight and embedded in paraffin and sectioned to a thickness of 5 μm. For immunohistochemistry, sections were deparaffinized, rehydrated, and endogenous peroxidase activity was blocked by incubating in 3% hydrogen peroxide. After antigen retrieval and antigen-blocking treatment, immunohistochemistry analyses were performed using a SPlink Detection Kits (Zhong Shan Jin Qiao) with specific antibodies overnight at 4°C. Negative controls were performed by incubation with non-immune IgGs. For immunofluorescence, secondary antibodies were changed to Alexa Fluor 488 goat anti-rabbit and Alexa Fluor 488 goat anti-mouse (Thermo Fisher Scientific), and the nuclei were counterstained with Hoechst 33342 for observation under a laser scanning confocal microscope (LSM 510 META, Zeiss, Germany). For immunocytometry, isolated mesenchymal progenitor cells were fixed with 4% PFA for CD24 and TCF21 staining. The antibodies used are as following, PCNA (CST #27214,USA), SDC4 (Proteintech #11820-1AP, China), ACTA2 (Proteintech # 23081-1-AP, China), CD44 (Proteintech # 60224-1-Ig, China), CD24 (Santa Cruz#sc-19585, USA), iNOS (Proteintech #22226, China), CD206 (Proteintech #18704), TCF21 (abcam #ab182134, USA), and Ki67 (Proteintech #28074-1-AP, China), TOP2A (Proteintech #20233-1-AP, China).

### Immunoblotting analysis

Ovarian proteins were extracted by radioimmunoprecipitation assay lysis buffer (P0013B, Beyotime Institute of Biotechnology, China) containing protease inhibitor cocktails (MCE, USA). Proteins were directly denatured with 5 × loading buffer. A total of 10 or 30 mg of proteins in each sample was loaded and separated by electrophoresis (165-8000, Bio-Rad, USA). After the electronic transfer (170-3930, Bio-Rad, USA) and blocking with 20 mL of 5% milk, the membranes were incubated overnight at 4°C with the following primary antibodies:P21 (Zenbio, #381102, China), mTOR (CST, #2983, USA), p-mTOR (Zenbio, #381548, China), SDC4 (Proteintech #11820-1AP, China), CD44 (Proteintech # 60224-1-Ig, China), p-RPS6 (9234, CST, USA), RPS6 (2217, CST, USA), p-AKT (9271, CST, USA), AKT (9272, CST, USA), β-Tubulin (Abbkine, China). After washing with Tris-buffered saline with Tween 20 (5 mL) three times, the horseradish peroxidase-conjugated relative secondary antibodies were then used to detect proteins through enhanced chemiluminescence (RPN2232, GE Healthcare, USA) on the Tanon 5200 analysis system.

### Follicle counting

Ovaries in the sham and injury groups were collected and fixed in 10% buffered formalin for 12 h, then embedded in paraffin and serially sectioned at a thickness of 5 μM for hematoxylin and eosin staining. All follicles with a visible nucleus were counted every second section [70]. Follicle classification was determined by Pederson’s system [71]: oocytes surrounded by a single layer of flattened or cuboidal granulosa cells were defined as primordial and primary follicles; oocytes surrounded by more than one layer of cuboidal granulosa cells with nonvisible antrum were determined to be secondary follicles. The antral follicle possessed a clearly defined antral space and a cumulus granulosa cell layer. Corpora lutea were filled with lutein cells, and follicles were considered atretic if they contained either a degenerating oocyte, disorganized granulosa cells, pyknotic nuclei, shrunken granulosa cells, or apoptotic bodies [70]. The results are reported as the percentage of each type of follicle per ovary.

### RT–PCR analysis

Total RNAs were isolated from ovaries by TRIzol reagent (Invitrogen). Then, 500–1000 ng RNA/per reaction were reverse transcribed into cDNAs using FastQuant RT Kit (TIANGEN Biotech, China). Quantitative RT–PCR was then performed using SYBR Green Mix (Applied Biological Materials, Canada) in an ABI StepOnePlus platform (Thermo Fisher Scientific). The primer sequences are listed in Table S12. The specificity of PCR products was assessed by melting curve analyses and amplicon size was determined by electrophoresis in 2% agarose gels.

### Masson staining

Ovarian sections at 5 μm thickness were used for staining with Masson solutions (Sbjbio life Sciences #BP-DL022) and images were captured using an Orthomorphic microscope (Nikon, Tokyo, Japan). The fibrosis area was quantified according to the previous report but with some modifications [56]. In brief, fibrosis was measured in a single section of the entire ovary using ImageJ software. Every image for analysis was first converted to a 16-bit image and changed colors with collagen staining as red and stroma as gray. A threshold was set on the basis of the staining in the ovaries of mice at 12M of age. While in the ovarian partial resection experiment, the staining in the ovaries of the sham group was set as the threshold. Fibrosis area was calculated as staining density compared to the stromal area.

### Statistics analysis

We explicitly described the statistical tools, methods, and threshold for each analysis of single-cell RNA sequencing data. Pearson’s correlation coefficients (*r*) were calculated to assess the link between the outgoing communication strength of the collagen pathway and aggregated outgoing communication strength. All the collected values are processed by GraphPad Prism 7.0 and Excel 2020 and presented as mean ± SD (standard deviation). One-way ANOVA analysis was used to determine significant differences between groups. All experiments were repeated at least three times, and a value of *P*-value < 0.05 was evaluated as statistically significant.

## Supplementary Material

lnae041_suppl_Supplementary_Table_S1

lnae041_suppl_Supplementary_Table_S2

lnae041_suppl_Supplementary_Table_S3

lnae041_suppl_Supplementary_Table_S4

lnae041_suppl_Supplementary_Table_S5

lnae041_suppl_Supplementary_Table_S6

lnae041_suppl_Supplementary_Table_S7

lnae041_suppl_Supplementary_Table_S8

lnae041_suppl_Supplementary_Table_S9

lnae041_suppl_Supplementary_Table_S10

lnae041_suppl_Supplementary_Table_S11

lnae041_suppl_Supplementary_Table_S12

lnae041_suppl_Supplementary_Figures_S1-S8

## Data Availability

The single-cell RNA-seq data used in this study were composed of two parts. One set of scRNA-seq data of mouse ovary from E11.5 to P5 were obtained from three recent studies [9, 11, 12], and were downloaded from Sequence Read Archive BioProject (accession number PRJNA562536, PRJNA528089, and PRJNA554804). The other set of scRNA-seq data of mouse ovary from P1, P14, W8, M8, and M12 was produced in our own laboratory and the sequence data have been deposited in the GEO database (GSE127106).
