# Supplementary material for: Single-cell RNA sequencing reveals the intercellular crosstalk and the regulatory landscape of stromal cells during the whole life of the mouse ovary
Source: Life Med. 2024 Dec 28;3(6):lnae041. doi: 10.1093/lifemedi/lnae041 (PMC11748273; doi:10.1093/lifemedi/lnae041)
Supplement: lnae041_suppl_Supplementary_Table_S12 [file lnae041_suppl_supplementary_table_s12.docx]

**Table S12 Real-Time PCR Primers**

| *Gene* | Forward primer 5’-3’ | Reverse primer 5’-3’ |
| --- | --- | --- |
| *Gapdh* | GCGGGCGCTGGAGGAA | GGATCTTCATGAGGTAGTCA |
| *Meg3* | CCTGCTGCCATCTACACCTC | CCTCTTCATCCTTTGCCATCCTGG |
| *Dlk1* | AAGGACTGCCAGAAAAAGGAC | GCAGAAATTGCCTGAGAAGC |
| *H19* | ACTCAGGAATCGGCTCTGGAA | CTGCTGTTCCGATGGTGTCTT |
| *Ptn* | CCATTTCCCTTCCGTTCC | AGGTTGCTACCGCTGAGTCC |
| *Igfbp5* | GGTTACCATGCGTAAGTCAAG | GCGTTGGAATCCTTGGAGT |
| *Peg3* | CTCACAACACAATCCAGGAC | TAGACCTCGACTGGTGCTTG |
| *Akr1cl* | TGCAGAGGTTCCTAAAAGTAAAGCTTTA | GGAAAATGAATAAGGTAGAGGTCAACATAA |
| *Cxcl14* | CGCTACAGCGACGTGAAGAA | GTTCCAGGCGTTGTACCAC |
| *Col1a1* | GATTCCCTGGACCTAAAGGTGC | AGCCTCTCCATCTTTGCCAGCA |
| *Col1a2* | GTGGCAGTGATGGAAGTGTG | AGGACCAGCGTTACCAACAG |
| *Col4a1* | CTAATGTCACAACATGGTGCTAC | GCAGGGTGTGTTAGTTACGC |
| *Col4a2* | TGGGACAGACGAGACAACAG | CAACGGTATTTGGGAGAACAT |
| *Col6a1* | AACCCCATGCAGTATAATGTCAC | AGGACCTGGTATTGAAGACGAG |
| *Col6a2* | AACGGATCGGCCCTCTGTG | GCCAGAGCTGTGTTATCCCAA |
| *Col6a3* | AAGCCTGTGTATCGTGGAG | TTAAGGCATTGGTCCCAAC |
| *Sdc4* | TTTGCCGTTTTCCTGATCCTG | TTGCCCAAGTCGTAACTGCC |
| *Sdc1* | AACGGGCCTCAACAGTCAG | CCGTGCGGATGAGATGTGA |
| *Birc5* | CTACCGAGAACGAGCCTGATT | AGCCTTCCAATTCCTTAAAGCAG |
| *Ki67* | ATCATTGACCGCTCCTTTAGGT | GCTCGCCTTGATGGTTCCT |
| *Mest* | GTGGTGGGTCCAAGTAGGG | AAGCACAACTATCTCAGGGCT |
| *Cks2* | TCGATGAGCACTACGAGTACC | CCATCCTAGACTCTGTTGGACAC |
| *Pclaf* | ACCAAAGCAAACTACGTTCCA | TTTTCCCGACGAACTTGAAGAA |
| *Smc2* | GGCTGGGATTACCAAAGCCTC | CACCAATAACCACCTGTCTTGT |
| *Stmn1* | TCTGTCCCCGATTTCCCCC | AGCTGCTTCAAGACTTCCGC |
| *Top2a* | CAACTGGAACATATACTGCTCCG | GGGTCCCTTTGTTTGTTATCAGC |
| *Ube2c* | CTCCGCCTTCCCTGAGTCA | GGTGCGTTGTAAGGGTAGCC |
| *Ccnb1* | AAGGTGCCTGTGTGTGAACC | GTCAGCCCCATCATCTGCG |
| *Nlrp3* | AGTCCTTGCAGGTCAGGTTC | TAGCAGTGAAGAGCAGTGCG |
| *Il-6* | TTCCATCCAGTTGCCTTCTTG | AATTAAGCCTCCGACTTGTGAA |
| *Il-1β* | ATGATGGCTTATTACAGTGGCAA | GTCGGAGATTCGTAGCTGGA |
| *iNOS* | ACCTTGTTCAGCTACGCCTT | TCTTCAGAGTCTGCCCATTG |
| *Arg-1* | CCAGAAGAATGGAAGAGTCAGT | GCAGATATGCAGGGAGTCACC |
| *Cd206* | TGATTACGAGCAGTGGAAGC | GTTCACCGTAAGCCCAATTT |
