# Supplementary material for: Single-cell RNA sequencing reveals the intercellular crosstalk and the regulatory landscape of stromal cells during the whole life of the mouse ovary
Source: Life Med. 2024 Dec 28;3(6):lnae041. doi: 10.1093/lifemedi/lnae041 (PMC11748273; doi:10.1093/lifemedi/lnae041)
Supplement: lnae041_suppl_Supplementary_Figures_S1-S8 [file lnae041_suppl_supplementary_figures_s1-s8.pdf]

Supplementary Materials for

Single-cell RNA sequencing reveals the intercellular crosstalk and the regulatory landscape of stromal cells during the whole life of the mouse ovary

Wan Jiang et al.

*Corresponding author. Email: Jing Li, E-mail: ljwth@njmu.edu.cn; Wenjie Shu, shuwj@bmi.ac.cn.

This PDF file includes:

Figs. S1 to S8

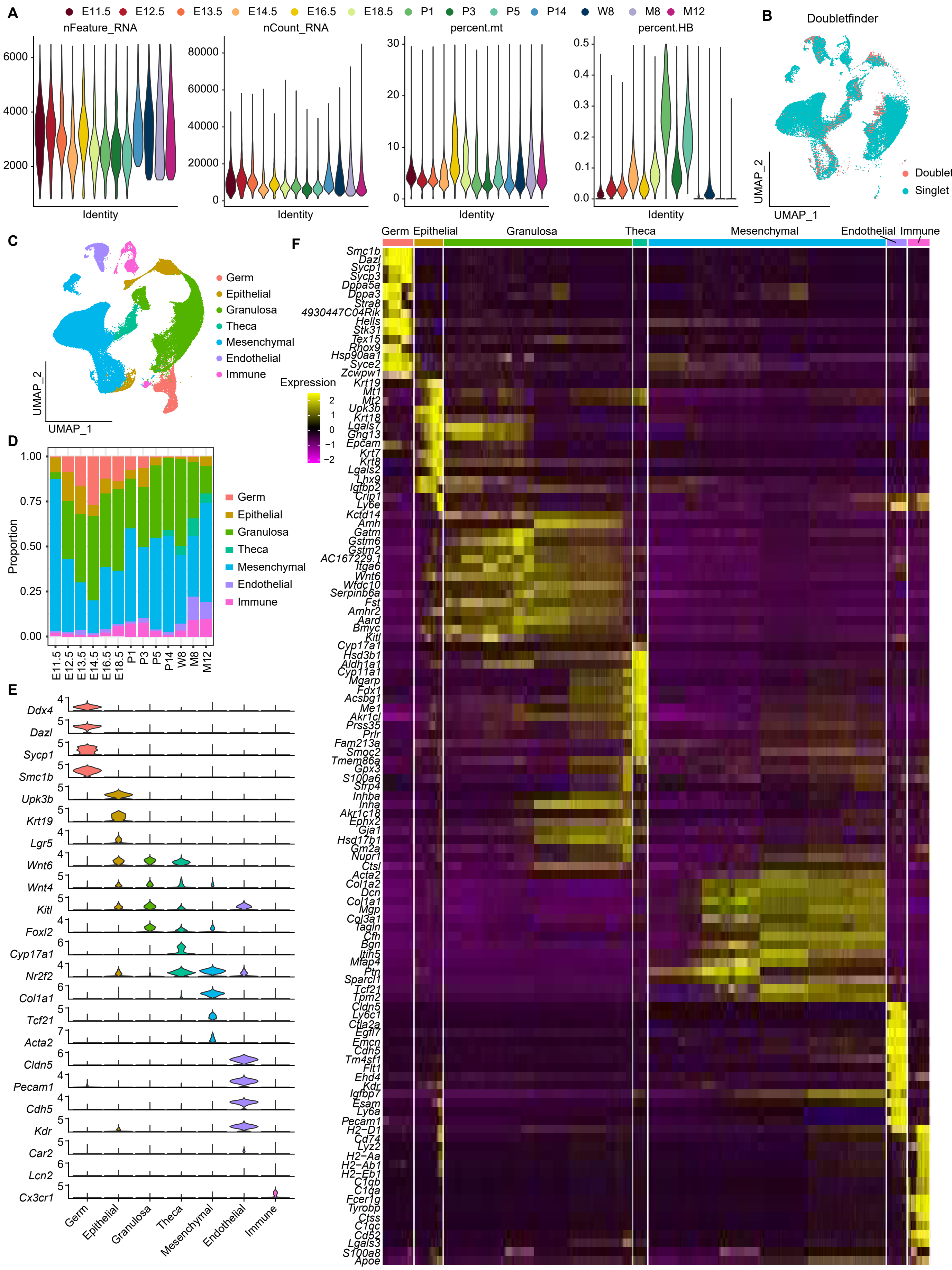
A
E11.5 E12.5 E13.5 E14.5 E16.5 E18.5 P1 P3 P5 P14 W8 M8 M12
nFeature_RNA
nCount_RNA
percent.mt
percent.HB
Identity
B
Doubletfinder
Doublet
Singlet
UMAP_1
UMAP_2
C
Germ
Epithelial
Granulosa
Theca
Mesenchymal
Endothelial
Immune
D
Proportion
E
Ddx4
Dazl
Sycp1
Smc1b
Upk3b
Krt19
Lgr5
Wnt6
Wnt4
Kitl
Foxl2
Cyp17a1
Nr2f2
Col1a1
Tcf21
Acta2
Cldn5
Pecam1
Cdh5
Kdr
Car2
Lcn2
Cx3cr1
F
Expression
Smc1b Dazl Sycp1 Sycp3 Dppa5a Dppa3 Stra8 4930447C04Rik Hells Stk31 Tex15 Rhox9 Hsp90aa1 Syce2 Zcwpw1 Krt19 Mt1 Mt2 Upk3b Krt18 Lgals7 Gng13 Epcam Krt7 Krt8 Lgals2 Lhx9 Igfbp2 Crip1 Ly6e Kctd14 Amh Gatm Gstm6 Gstm2 AC167229.1 Itga6 Wnt6 Wfdc10 Serpinb6a Fst Amhr2 Aard Bmyc Kitl Cyp17a1 Hsd3b1 Aldh1a1 Cyp11a1 Mgarp Fdx1 Acsbg1 Me1 Akr1cl Prss35 Prlr Fam213a Smoc2 Tmem86a Gpx3 S100a6 Sfrp4 Inhba Inha Akr1c18 Ephx2 Gja1 Hsd17b1 Gm2a Nupr1 Ctsl Acta2 Col1a2 Dcn Col1a1 Mgp Col3a1 Tagln Cfh Bgn Itih5 Mfap4 Ptn Sparcl1 Tcf21 Tpm2 Cldn5 Ly6c1 Ctla2a Egfl7 Emcn Cdh5 Tm4sf1 Flt1 Ehd4 Kdr Igfbp7 Esam Ly6a Pecam1 H2-D1 Cd74 Lyz2 H2-Aa H2-Ab1 H2-Eb1 C1qb C1qa Fcer1g Tyrobp Ctss C1qc Cd52 Lgals3 S100a8 Apoe

Figure S1. Quality control and cell type characterization of data sets.

(A) Violin diagram shows the number of unique genes (nFeature, the total number of molecules (nCount), the percentage of mitochondrial genes (percent.mt) and the percentage of erythrocyte genes (percent.HB) in different data sets. (B) UMAP visualization of doublets (red) among data sets. (C) UMAP plot of the single cell distribution in mouse ovary, coloured by the identified 7 cell clusters. (D) Cell number distributions of 7 cell clusters from E11.5 to M12 of ovaries. (E) Violin plot of marker gene expression in 7 cell clusters. (F) Heatmap of top 15 differentially expressed genes in each cell cluster.

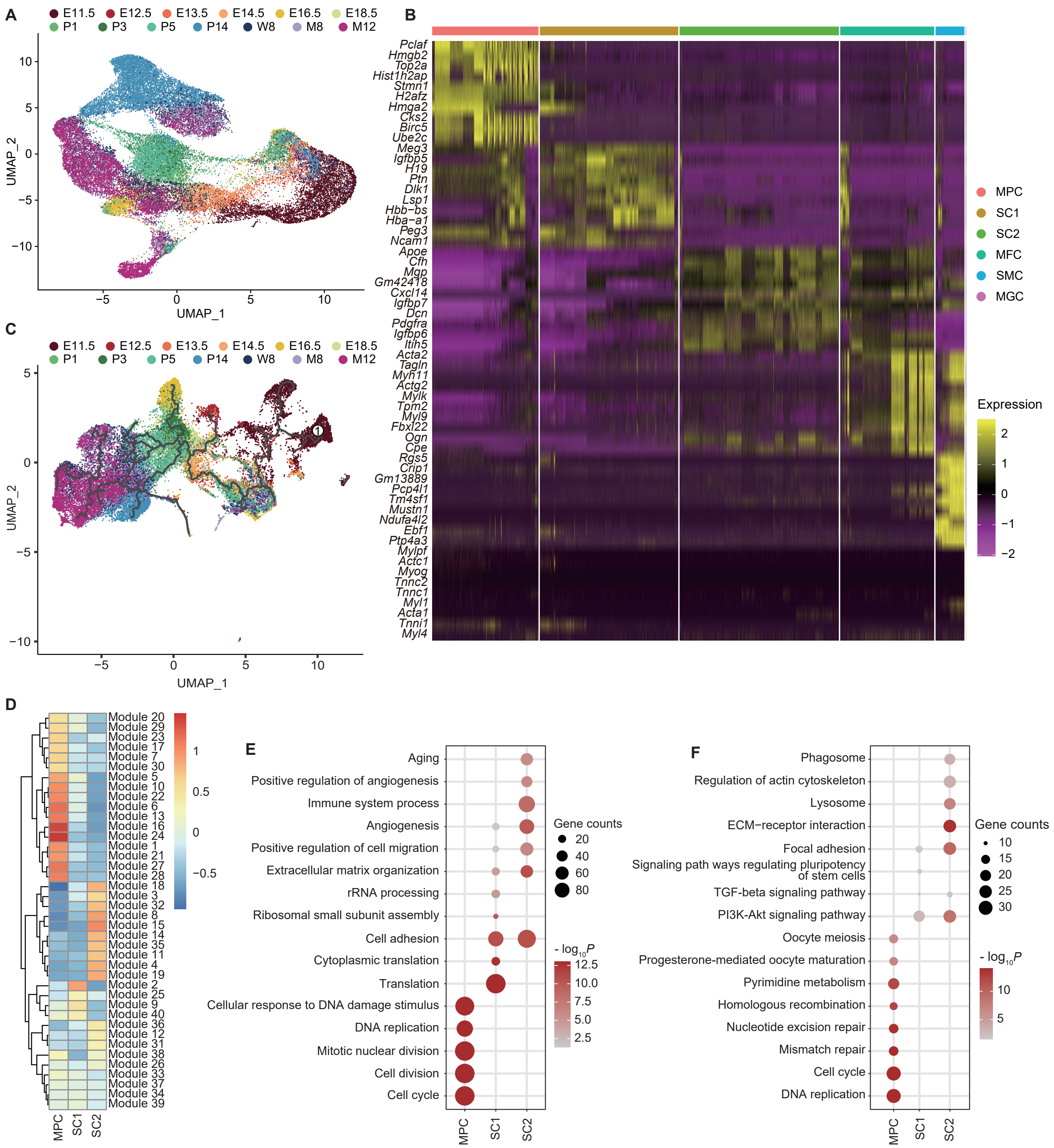
A
E11.5 E12.5 E13.5 E14.5 E16.5 E18.5
P1 P3 P5 P14 W8 M8 M12
UMAP_1
UMAP_2
B
Pclaf
Hmgb2
Top2a
Hist1h2ap
Stmn1
H2afz
Hmga2
Cks2
Birc5
Ube2c
Meg3
Igfbp5
H19
Ptn
Dlk1
Lsp1
Hbb−bs
Hba−a1
Peg3
Ncam1
Apoe
Cfh
Mgp
Gm42418
Cxcl14
Igfbp7
Dcn
Pdgfra
Igfbp6
Itih5
Acta2
Tagln
Myh11
Actg2
Mylk
Tpm2
Myl9
Fbxl22
Ogn
Cpe
Rgs5
Crip1
Gm13889
Pcp4l1
Tm4sf1
Mustn1
Ndufa4l2
Ebf1
Ptp4a3
Mylpf
Actc1
Myog
Tnnc2
Tnnc1
Myl1
Acta1
Tnni1
Myl4
MPC
SC1
SC2
MFC
SMC
MGC
Expression
C
D
Module 20
Module 29
Module 23
Module 17
Module 7
Module 30
Module 5
Module 10
Module 22
Module 6
Module 13
Module 16
Module 24
Module 1
Module 21
Module 27
Module 28
Module 18
Module 3
Module 32
Module 8
Module 15
Module 14
Module 35
Module 11
Module 4
Module 19
Module 2
Module 25
Module 9
Module 40
Module 36
Module 12
Module 31
Module 38
Module 26
Module 33
Module 37
Module 34
Module 39
MPC SC1 SC2
E
Aging
Positive regulation of angiogenesis
Immune system process
Angiogenesis
Positive regulation of cell migration
Extracellular matrix organization
rRNA processing
Ribosomal small subunit assembly
Cell adhesion
Cytoplasmic translation
Translation
Cellular response to DNA damage stimulus
DNA replication
Mitotic nuclear division
Cell division
Cell cycle
Gene counts
20
40
60
80
- log10P
12.5
10.0
7.5
5.0
2.5
F
Phagosome
Regulation of actin cytoskeleton
Lysosome
ECM−receptor interaction
Focal adhesion
Signaling path ways regulating pluripotency of stem cells
TGF-beta signaling pathway
PI3K-Akt signaling pathway
Oocyte meiosis
Progesterone-mediated oocyte maturation
Pyrimidine metabolism
Homologous recombination
Nucleotide excision repair
Mismatch repair
Cell cycle
DNA replication
Gene counts
10
15
20
25
30
- log10P
10
5

Figure S2. Dynamics of mesenchymal cells in the whole life of ovary.

(A) UMAP plot of mesenchymal cell distribution coloured by developmental stage from E11.5 to M12. (B) Heatmap of top 10 differentially expressed genes in mesenchymal cell subsets. (C) UMAP visualization of the cell trajectory from mesenchymal progenitor cell (MPC) to stroma 1 (SC1) and stroma 2 (SC2) plotted by developmental stage from E11.5 to M12. (D) Heatmap of pseudotime-related gene modules of stromal cell subpopulations, red to blue, represent the average expression level of the gene module from high to low. (E, F) GO and KEGG pathway enrichment analysis of pseudotime-related gene modules in stromal cell types (MPC, SC1 and SC2).

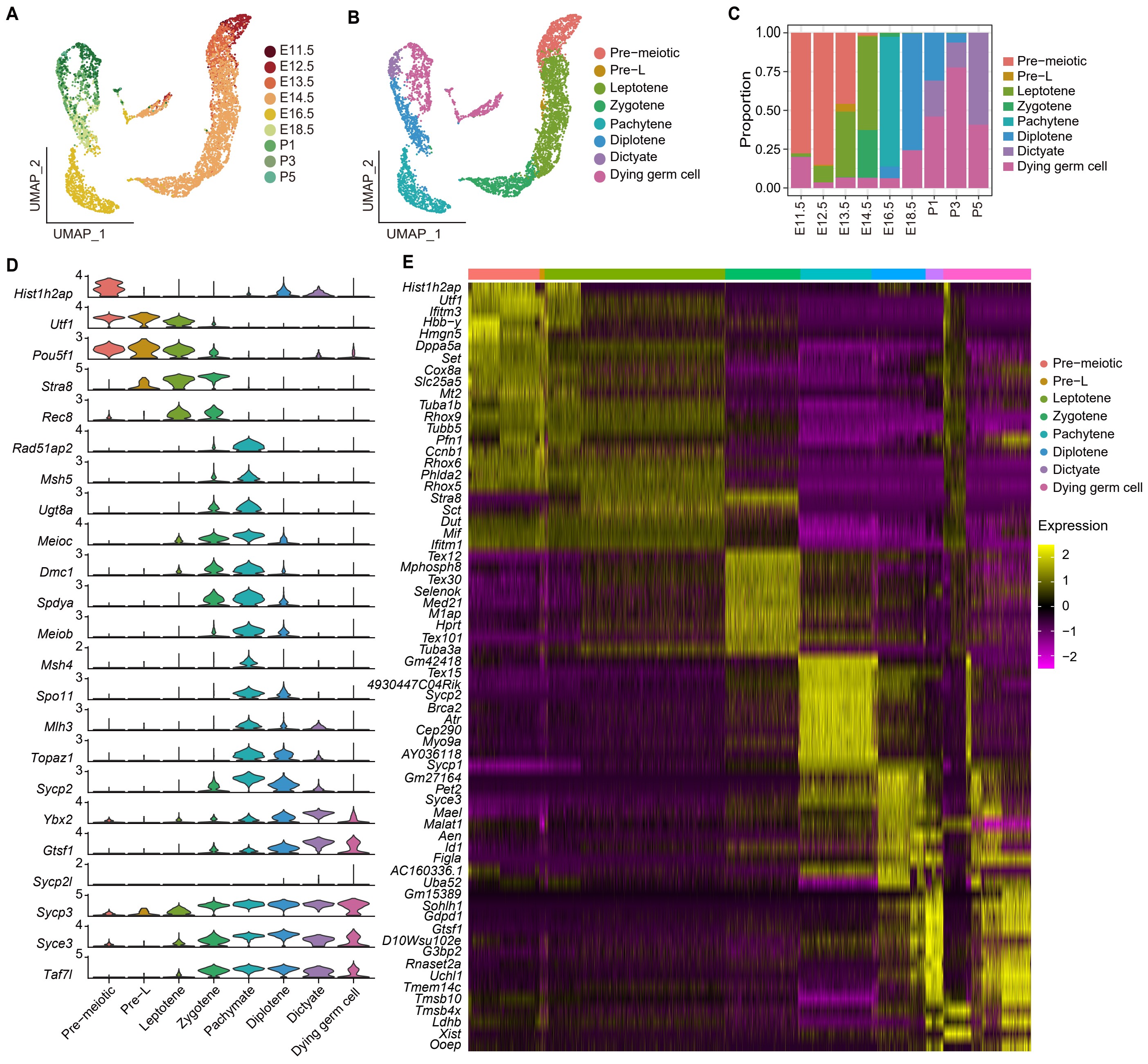

A
E11.5
E12.5
E13.5
E14.5
E16.5
E18.5
P1
P3
P5
UMAP_2
UMAP_1
B
Pre−meiotic
Pre−L
Leptotene
Zygotene
Pachytene
Diplotene
Dictyate
Dying germ cell
C
Proportion
1.00
0.75
0.50
0.25
0.00
D
Hist1h2ap
Utf1
Pou5f1
Stra8
Rec8
Rad51ap2
Msh5
Ugt8a
Meioc
Dmc1
Spdya
Meiob
Msh4
Spo11
Mlh3
Topaz1
Sycp2
Ybx2
Gtsf1
Sycp2l
Sycp3
Syce3
Taf7l
Pachymate
E
Expression

Figure S3. Dynamics of germ cells during ovarian development.

(A) UMAP plot of germ cell distribution, coloured by developmental stage from E11.5 to P5. (B) UMAP plot of germ cell distribution, coloured according to 8 identified germ cell subgroups. (C) Cell number distributions of 8 germ cell subgroups from E11.5 to P5. Violin plot of marker gene expression in 8 germ cell subpopulations. (E) Heatmap of top 10 differentially expressed genes in germ cell subpopulations.

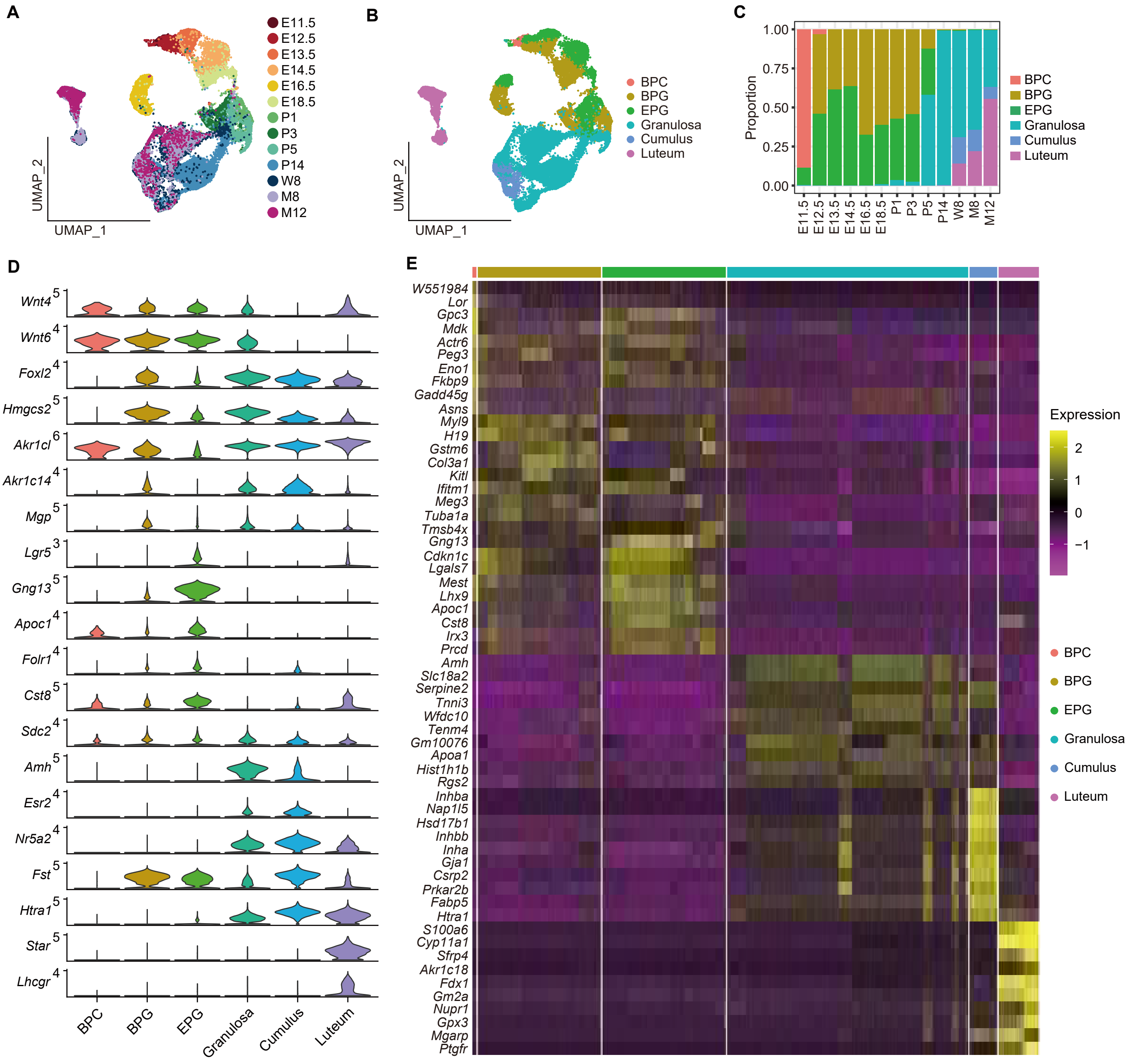

A
E11.5
E12.5
E13.5
E14.5
E16.5
E18.5
P1
P3
P5
P14
W8
M8
M12
UMAP_2
UMAP_1
B
BPC
BPG
EPG
Granulosa
Cumulus
Luteum
C
Proportion
1.00
0.75
0.50
0.25
0.00
D
Wnt4
Wnt6
Foxl2
Hmgcs2
Akr1cl
Akr1c14
Mgp
Lgr5
Gng13
Apoc1
Folr1
Cst8
Sdc2
Amh
Esr2
Nr5a2
Fst
Htra1
Star
Lhcgr
E
W551984
Lor
Gpc3
Mdk
Actr6
Peg3
Eno1
Fkbp9
Gadd45g
Asns
Myl9
H19
Gstm6
Col3a1
Kitl
Ifitm1
Meg3
Tuba1a
Tmsb4x
Gng13
Cdkn1c
Lgals7
Mest
Lhx9
Apoc1
Cst8
Irx3
Prcd
Amh
Slc18a2
Serpine2
Tnni3
Wfdc10
Tenm4
Gm10076
Apoa1
Hist1h1b
Rgs2
Inhba
Nap1l5
Hsd17b1
Inhbb
Inha
Gja1
Csrp2
Prkar2b
Fabp5
Htra1
S100a6
Cyp11a1
Sfrp4
Akr1c18
Fdx1
Gm2a
Nupr1
Gpx3
Mgarp
Ptgfr
Expression

Figure S4. Dynamics of granulosa cells subclusters in the whole life of ovary.

(A) UMAP plot of granulosa cell distribution, coloured by developmental stage from E11.5 to M12. (B) UMAP plot of granulosa cell distribution from E11.5 to M12, colored by 6 identified granulosa cell subgroups: bipotential cell (BPC); bipotential pregranulosa cells (BPG); epithelial pregranulosa cells (EPG); granulosa cells; cumulus cell; luteum cells. (C) Cell number distributions of 6 granulosa subgroups from E11.5 to M12. (D) Violin plot of marker gene expression in 6 granulosa cell subpopulations. (E) Heatmap of top 10 differentially expressed genes in granulosa cell subpopulations.

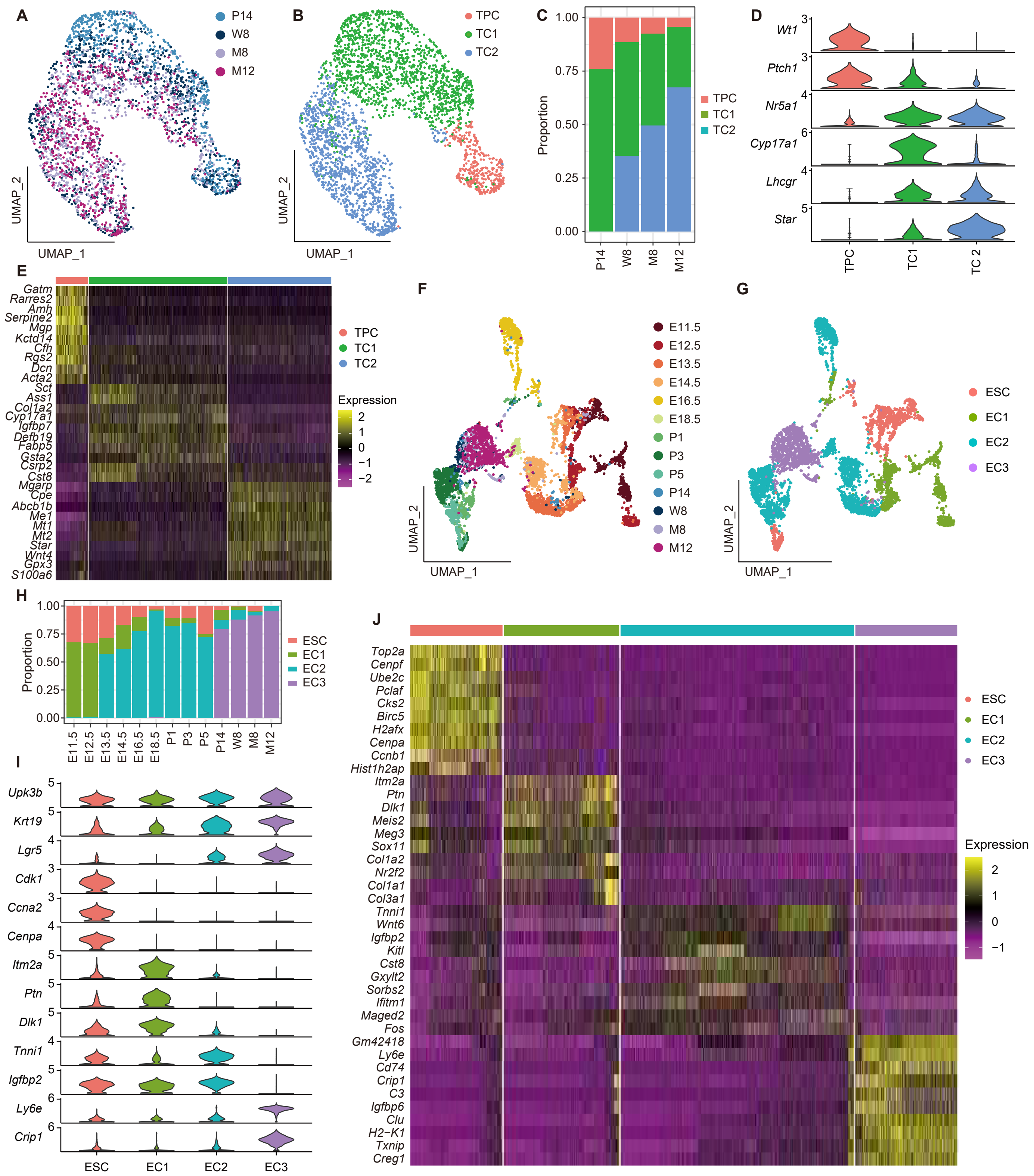

A
P14
W8
M8
M12
UMAP_1
UMAP_2
B
TPC
TC1
TC2
C
Proportion
TPC
TC1
TC2
D
Wt1
Ptch1
Nr5a1
Cyp17a1
Lhcgr
Star
E
Gatm
Rarres2
Amh
Serpine2
Mgp
Kctd14
Cfh
Rgs2
Dcn
Acta2
Sct
Ass1
Col1a2
Cyp17a1
Igfbp7
Defb19
Fabp5
Gsta2
Csrp2
Cst8
Mgarp
Cpe
Abcb1b
Me1
Mt1
Mt2
Star
Wnt4
Gpx3
S100a6
Expression
F
E11.5
E12.5
E13.5
E14.5
E16.5
E18.5
P1
P3
P5
G
ESC
EC1
EC2
EC3
H
I
Upk3b
Krt19
Lgr5
Cdk1
Ccna2
Cenpa
Itm2a
Ptn
Dlk1
Tnni1
Igfbp2
Ly6e
Crip1
J
Top2a
Cenpf
Ube2c
Pclaf
Cks2
Birc5
H2afx
Ccnb1
Hist1h2ap
Meis2
Meg3
Sox11
Nr2f2
Col1a1
Col3a1
Wnt6
Kitl
Gxylt2
Sorbs2
Ifitm1
Maged2
Fos
Gm42418
Cd74
C3
Igfbp6
Clu
H2−K1
Txnip
Creg1

Figure S5. Dynamics of theca cell and epithelial cell subclusters during ovarian development.

(A) UMAP plot of theca cell distribution, coloured by developmental stage from D14 to M12. (B) UMAP plot of theca cell distribution from D14 to M12, colored by 3 identified theca cell subgroups: theca progenitor cell (TPC); type 1 theca cell (TC1); type 2 theca cell (TC2). (C) Cell number distributions of 3 subgroups from D14 to M12. (D) Violin plot of marker gene expression in 3 theca cell subpopulations. (E) Heatmap of top 10 differentially expressed genes in theca cell subpopulations. (F) UMAP plot of epithelial cell distribution, coloured by developmental stage from E11.5 to M12. (G) UMAP plot of epithelial cell from E11.5 to M12, colored by 4 identified epithelial cell subgroups: epithelial stem cell (ESC); type 1 epithelial cell (EC1); type 2 epithelial cell (EC2); type 3 epithelial cell (EC3). (H) Cell number distribution of 4 epithelial subgroups from E11.5 to M12. (I) Violin plot of marker gene expression in 4 epithelial cell subpopulations. (J) Heatmap of top 10 differentially expressed genes in epithelial cell subpopulations.

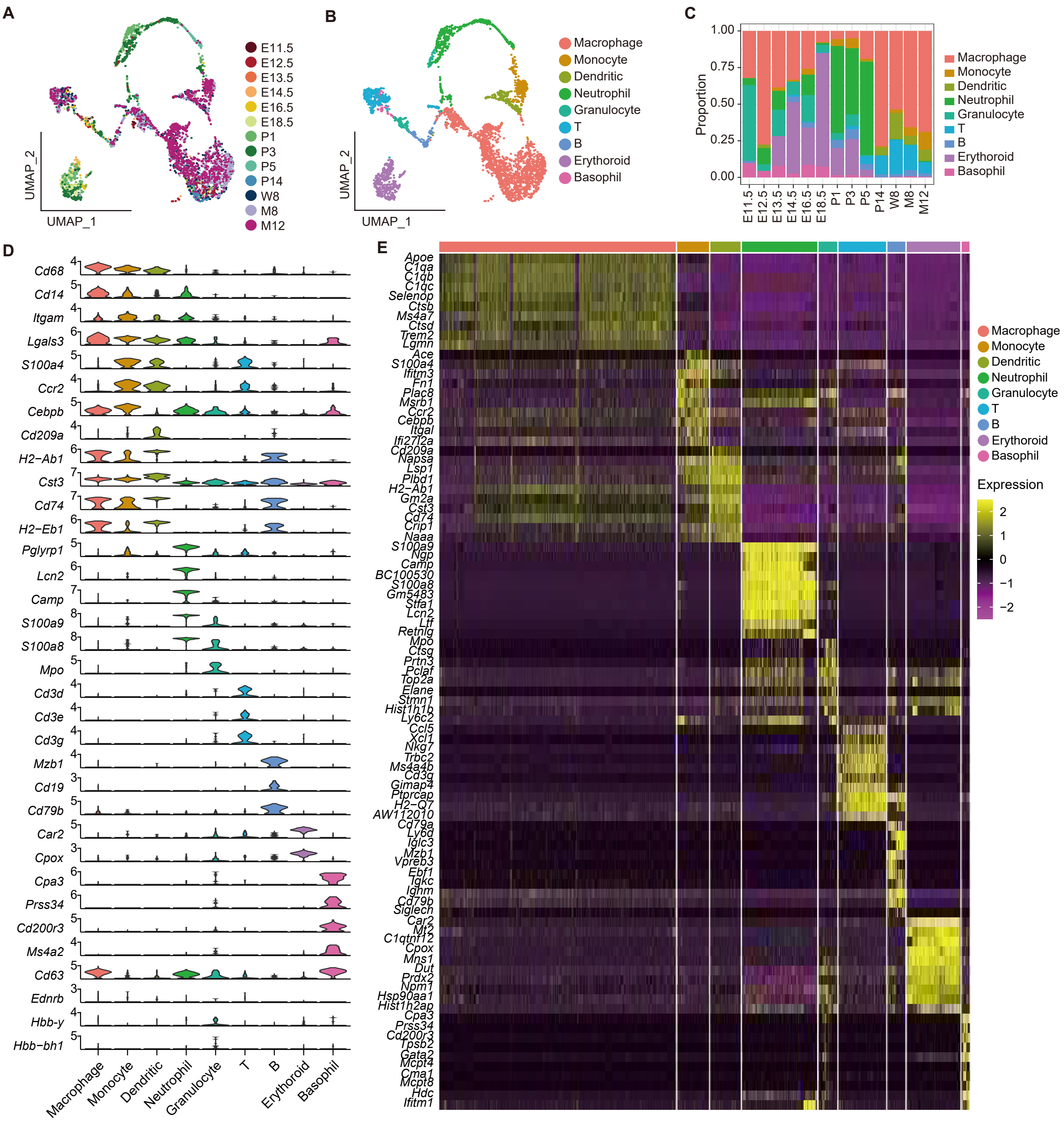
A
B
C
D
E
UMAP_1
UMAP_2
E11.5
E12.5
E13.5
E14.5
E16.5
E18.5
P1
P3
P5
P14
W8
M8
M12
Macrophage
Monocyte
Dendritic
Neutrophil
Granulocyte
T
B
Erythoroid
Basophil
Proportion
1.00
0.75
0.50
0.25
0.00
Cd68
Cd14
Itgam
Lgals3
S100a4
Ccr2
Cebpb
Cd209a
H2−Ab1
Cst3
Cd74
H2−Eb1
Pglyrp1
Lcn2
Camp
S100a9
S100a8
Mpo
Cd3d
Cd3e
Cd3g
Mzb1
Cd19
Cd79b
Car2
Cpox
Cpa3
Prss34
Cd200r3
Ms4a2
Cd63
Ednrb
Hbb−y
Hbb−bh1
Expression
2
1
0
−1
−2

Figure S6. Dynamic changes of immune cell subclusters in the whole life of ovary.

(A) UMAP plot of immune cell distribution, coloured by developmental stage from E11.5 to M12. (B) UMAP plot of immune cell distribution, coloured according to 9 identified immune cell subgroups. (C) Cell number distributions of 9 immune cell subclusters from E11.5 to M12. (D) Violin plot of marker gene expression in 9 immune cell subpopulations. (E) Heatmap of top 10 differentially expressed genes in immune cell subpopulations.

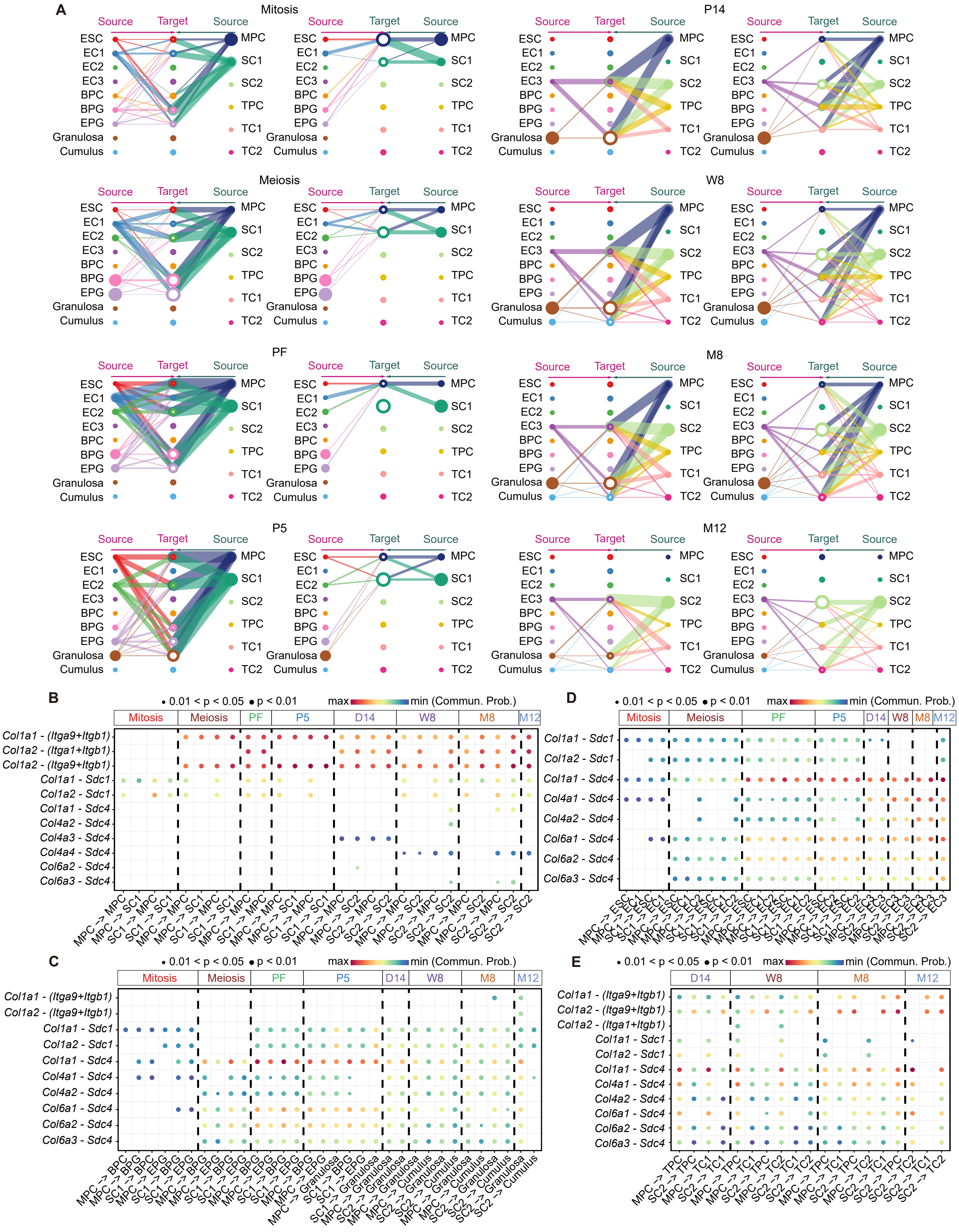
A
Mitosis
Meiosis
PF
P5
P14
W8
M8
M12
Source
Target
ESC
EC1
EC2
EC3
BPC
BPG
EPG
Granulosa
Cumulus
MPC
SC1
SC2
TPC
TC1
TC2
B
C
D
E
0.01 < p < 0.05
p < 0.01
max
min (Commun. Prob.)
D14
Col1a1 - (Itga9+Itgb1)
Col1a2 - (Itga1+Itgb1)
Col1a2 - (Itga9+Itgb1)
Col1a1 - Sdc1
Col1a2 - Sdc1
Col1a1 - Sdc4
Col4a1 - Sdc4
Col4a2 - Sdc4
Col4a3 - Sdc4
Col4a4 - Sdc4
Col6a1 - Sdc4
Col6a2 - Sdc4
Col6a3 - Sdc4

Figure S7. Identification of ligand-receptor pairs of collagens.

(A) Hierarchical plot of intercellular communication networks for collagen signaling during mouse ovary development stages. This plot consists of two parts: left and right portions highlight the subpopulations of epithelial, granulosa cells as targets, and subpopulations of stromal, theca cells as targets, respectively. Solid and open circles represent source and target, respectively. Circle size, edge width, and edge color represent cell numbers, communication strength, and signaling source, respectively. (B–E) The significant ligand-receptor pairs ($P < 0.05$) that contribute to the collagen signaling pathway sending from stromal subpopulations to stromal subpopulations (B), granulosa subpopulations (C), epithelial subpopulations (D), and theca subpopulations (E). The dot color and size represent the calculated communication probability and p-value, respectively. *p*-values are derived from one-sided permutation test.

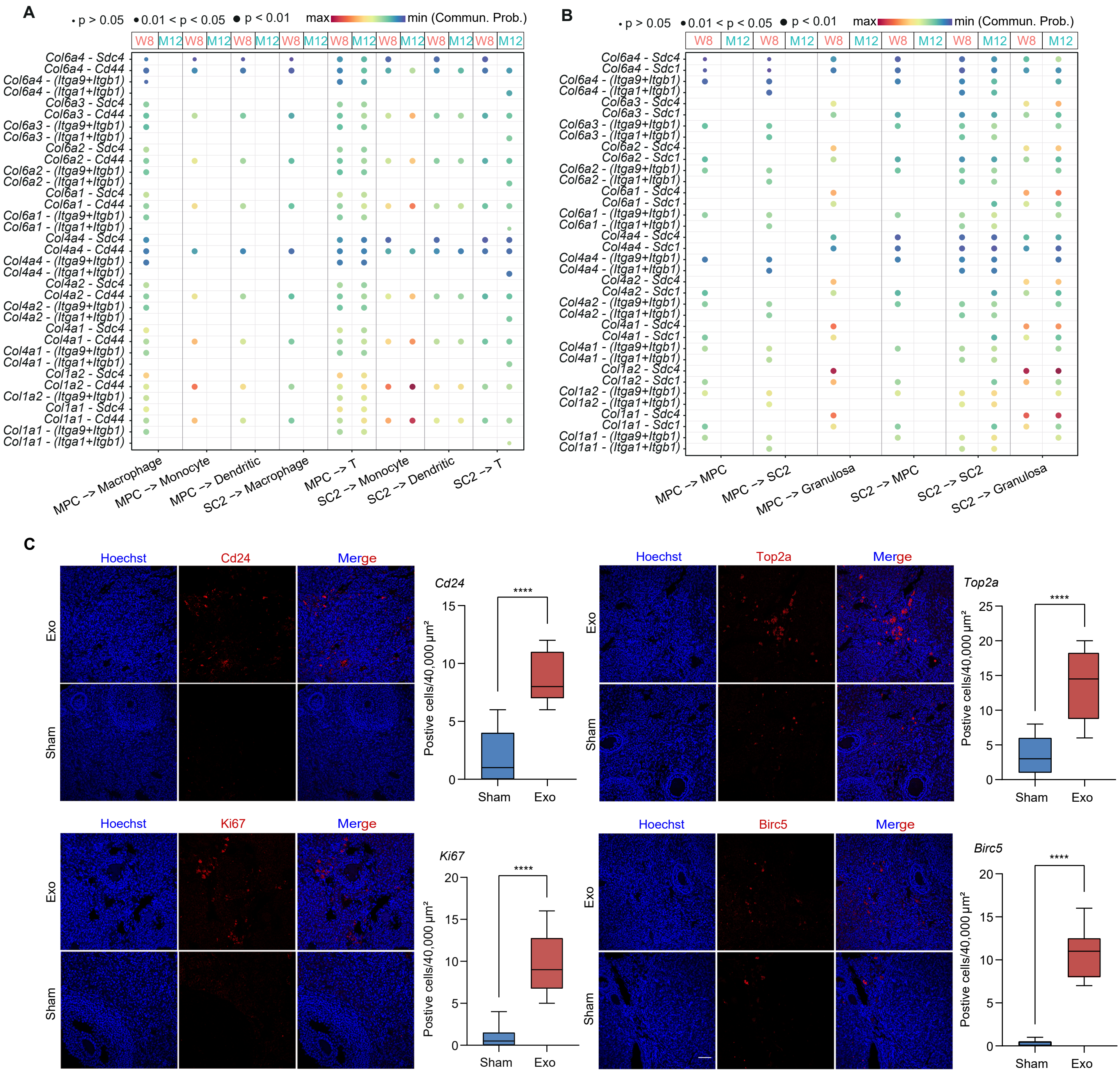
A
• p > 0.05 • 0.01 < p < 0.05 ● p < 0.01
max min (Commun. Prob.)
W8 M12 W8 M12 W8 M12 W8 M12 W8 M12 W8 M12 W8 M12 W8 M12
Col6a4 - Sdc4
Col6a4 - Cd44
Col6a4 - (Itga9+Itgb1)
Col6a4 - (Itga1+Itgb1)
Col6a3 - Sdc4
Col6a3 - Cd44
Col6a3 - (Itga9+Itgb1)
Col6a3 - (Itga1+Itgb1)
Col6a2 - Sdc4
Col6a2 - Cd44
Col6a2 - (Itga9+Itgb1)
Col6a2 - (Itga1+Itgb1)
Col6a1 - Sdc4
Col6a1 - Cd44
Col6a1 - (Itga9+Itgb1)
Col6a1 - (Itga1+Itgb1)
Col4a4 - Sdc4
Col4a4 - Cd44
Col4a4 - (Itga9+Itgb1)
Col4a4 - (Itga1+Itgb1)
Col4a2 - Sdc4
Col4a2 - Cd44
Col4a2 - (Itga9+Itgb1)
Col4a2 - (Itga1+Itgb1)
Col4a1 - Sdc4
Col4a1 - Cd44
Col4a1 - (Itga9+Itgb1)
Col4a1 - (Itga1+Itgb1)
Col1a2 - Sdc4
Col1a2 - Cd44
Col1a2 - (Itga9+Itgb1)
Col1a1 - Sdc4
Col1a1 - Cd44
Col1a1 - (Itga9+Itgb1)
Col1a1 - (Itga1+Itgb1)
MPC -> Macrophage
MPC -> Monocyte
MPC -> Dendritic
SC2 -> Macrophage
MPC -> T
SC2 -> Monocyte
SC2 -> Dendritic
SC2 -> T
B
• p > 0.05 • 0.01 < p < 0.05 ● p < 0.01
max min (Commun. Prob.)
W8 M12 W8 M12 W8 M12 W8 M12 W8 M12 W8 M12
Col6a4 - Sdc4
Col6a4 - Sdc1
Col6a4 - (Itga9+Itgb1)
Col6a4 - (Itga1+Itgb1)
Col6a3 - Sdc4
Col6a3 - Sdc1
Col6a3 - (Itga9+Itgb1)
Col6a3 - (Itga1+Itgb1)
Col6a2 - Sdc4
Col6a2 - Sdc1
Col6a2 - (Itga9+Itgb1)
Col6a2 - (Itga1+Itgb1)
Col6a1 - Sdc4
Col6a1 - Sdc1
Col6a1 - (Itga9+Itgb1)
Col6a1 - (Itga1+Itgb1)
Col4a4 - Sdc4
Col4a4 - Sdc1
Col4a4 - (Itga9+Itgb1)
Col4a4 - (Itga1+Itgb1)
Col4a2 - Sdc4
Col4a2 - Sdc1
Col4a2 - (Itga9+Itgb1)
Col4a2 - (Itga1+Itgb1)
Col4a1 - Sdc4
Col4a1 - Sdc1
Col4a1 - (Itga9+Itgb1)
Col4a1 - (Itga1+Itgb1)
Col1a2 - Sdc4
Col1a2 - Sdc1
Col1a2 - (Itga9+Itgb1)
Col1a2 - (Itga1+Itgb1)
Col1a1 - Sdc4
Col1a1 - Sdc1
Col1a1 - (Itga9+Itgb1)
Col1a1 - (Itga1+Itgb1)
MPC -> MPC
MPC -> SC2
MPC -> Granulosa
SC2 -> MPC
SC2 -> SC2
SC2 -> Granulosa
C
Hoechst
Cd24
Merge
Exo
Sham
Cd24
****
Postive cells/40,000 μm²
0
5
10
15
Sham
Exo
Hoechst
Top2a
Merge
Exo
Sham
Top2a
****
Postive cells/40,000 μm²
0
5
10
15
20
25
Sham
Exo
Hoechst
Ki67
Merge
Exo
Sham
Ki67
****
Postive cells/40,000 μm²
0
5
10
15
20
Sham
Exo
Hoechst
Birc5
Merge
Exo
Sham
Birc5
****
Postive cells/40,000 μm²
0
5
10
15
20
Sham
Exo

Figure S8. Comparison analysis of collagen signaling pathway between W8 and M12 ovaries. (A) Comparison of communication probabilities mediated by ligand-receptor pairs from stromal subgroups to immune subgroups between W8 and M12 ovaries. (B) Comparison of communication probabilities mediated by ligand-receptor pairs from stromal subgroups to stromal subgroups and granulosa cells between W8 and M12 ovaries. (C) Immunofluorescence showing an increase in the number of MPCs in the Exosomes injection groups at 21 days after ovarian injection in situ in 10M mice. The data represent the results (mean ± SD) of the biological triplicate experiments. **** $P <$ 0.0001, by two-tailed unpaired Student -test. Bars = 50 μm.
